# Human T-cell lymphotropic virus type 1 (HTLV-1) proposed vaccines: a systematic review of preclinical and clinical studies

**DOI:** 10.1186/s12879-023-08289-7

**Published:** 2023-05-11

**Authors:** Niloofar Seighali, Arman Shafiee, Mohammad Ali Rafiee, Dlnya Aminzade, Sayed-Hamidreza Mozhgani

**Affiliations:** 1 Student Research Committee, School of Medicine, Alborz University of Medical Sciences, Karaj, Iran; 2 School of Medicine, Shahid Beheshti University of Medical Sciences, Tehran, Iran; 3 Department of Microbiology, School of Medicine, Alborz University of Medical Sciences, Karaj, Iran; 4 Non-Communicable Disease Research Center, Alborz University of Medical Sciences, Karaj, Iran

**Keywords:** HTLV-1, Vaccine

## Abstract

**Background:**

Numerous vaccination research experiments have been conducted on non-primate hosts to prevent or control HTLV-1 infection. Therefore, reviewing recent advancements for status assessment and strategic planning of future preventative actions to reduce HTLV-1 infection and its consequences would be essential.

**Methods:**

MEDLINE, Scopus, Web of Science, and Clinicaltrials.gov were searched from each database's inception through March 27, 2022. All original articles focusing on developing an HTLV-1 vaccine candidate were included.

**Results:**

A total of 47 studies were included. They used a variety of approaches to develop the HTLV-1 vaccine, including DNA-based, dendritic-cell-based, peptide/protein-based, and recombinant vaccinia virus approaches. The majority of the research that was included utilized Tax, Glycoprotein (GP), GAG, POL, REX, and HBZ as their main peptides in order to develop the vaccine. The immunization used in dendritic cell-based investigations, which were more recently published, was accomplished by an activated CD-8 T-cell response. Although there hasn't been much attention lately on this form of the vaccine, the initial attempts to develop an HTLV-1 immunization depended on recombinant vaccinia virus, and the majority of results seem positive and effective for this type of vaccine. Few studies were conducted on humans. Most of the studies were experimental studies using animal models. Adenovirus, Cytomegalovirus (CMV), vaccinia, baculovirus, hepatitis B, measles, and pox were the most commonly used vectors.

**Conclusions:**

This systematic review reported recent progression in the development of HTLV-1 vaccines to identify candidates with the most promising preventive and therapeutic effects.

**Supplementary Information:**

The online version contains supplementary material available at 10.1186/s12879-023-08289-7.

## Introduction

Human T-cell lymphotropic virus type 1 (HTLV-1) is a member of the *Deltaretrovirus* genus. The number of infected individuals is currently estimated at approximately 5–10 million globally [1–3]. The main endemic areas are Japan, sub-Saharan Africa, South America, the Caribbean area, Iran, Romania, and Melanesia [4–6]. HTLV-1 is capable of inducing or strongly associated with several serious medical conditions such as adult T-cell leukemia/lymphoma (ATLL), HTLV-1-associated myelopathy/tropical spastic paraparesis (HAM/TSP), and a variety of inflammatory processes, including uveitis and dermatitis [1, 7]. Furthermore, evidence suggests HTLV-1 links to bronchitis, Sjögren’s syndrome, rheumatoid arthritis, fibromyalgia, and ulcerative colitis [8]. Although only 3–5% of seropositive individuals develop ATLL throughout their life, consequences can enormously impact the individual. Although there are less aggressive forms of ATLL such as the smoldering form, ATLL is frequently considered a highly aggressive poor prognosis form of non-Hodgkin's lymphoma accompanied by generalized lymphadenopathy, skin lesions, hepatosplenomegaly, and hypercalcemia. HAM/TSP characterized by an insidious onset of progressive weakness of lower limbs, urinary/bowel dysfunction, and lumbar pain affects approximately 0.25–3.7% of HTLV-1 carriers based on ethnic susceptibility [1, 9–12]. Notably, High previous load appears to be a risk factor for developing both ATL and HAM/TSP in infected HTLV-1 individuals [2, 13, 14].

Upon direct contact via cell-containing body fluids, including blood, breast milk, and semen, the transmission of HTLV-1 is possible [15]. In addition, the main risk factors among these transmission routes include but are not limited to the exposure time (e.g., duration of breastfeeding), HTLV-1 provincial load in blood or milk, and HLA compatibility. At the molecular level, transmission is described by binding to receptors like glucose transporter 1 (Glut-1), neuropilin-1 (NRP-1), and heparan sulfate proteoglycans (HSPG) [16–19]. To raise the number of infected cells, HTLV-1 alters the immunophenotypic features of infected cells and then uses the combined action of HTLV-1 bZIP factor and Tax to inhibit apoptosis and induce proliferation [20].

Since the detection and isolation of HTLV-1 by Robert C. Gallo et al. in 1980 [21], no proven measure to cure HTLV-1 infection nor any effective therapeutic management to alter the poor prognosis of patients with ATL has been announced [10]. Moreover, the clinical management of HAM/TSP is still challenging and particularly unsatisfactory [11]. Additionally, underestimation of the total number of infected individuals is not improbable due to a lack of data [4, 6]. Accordingly, the implantation of preventive measures such as screening or vaccination to lessen the cumulative burden of this pathogenic agent is considered crucial [22, 23]. Currently, there are several suggested preventive measures namely antenatal screening and screening of blood and organ donors [24–27]. However, there is debate about the cost-effectiveness of these measures mainly based on the heterogeneity of endemicity of infection in different geographical areas [22, 28]. The production of an efficacious safe vaccine would prevent the transmission of HTLV-1 and in case of a reduction in proviral load in infected individuals can even lead to less probability to develop HTLV-1 linked diseases.

Numerous vaccine research studies including recombinant peptide or protein, naked DNA, and antibodies have been carried out, mostly on non-primate hosts. Therefore, an evaluation of recent progress in this field would be beneficial for status assessment and strategic planning of future preventive measures to diminish HTLV-1 infection and its outcomes. Taking these into consideration, we aimed to systematically review developed HTLV-1 vaccines by appraising the available literature.

## Methods

This study is reported based on the Preferred Reporting Items for Systematic Reviews and Meta-Analyses (PRISMA) guidelines.

### Search strategy

We have searched Medline (through PubMed), Scopus, and Web of Science until March 27, 2022. The following keywords were searched to retrieve relevant studies: (((("Human T-lymphotropic virus 1"[Mesh]) OR (Human T-lymphotropic virus 1[Title/Abstract])) OR (Human T lymphotropic virus 1[Title/Abstract])) OR (HTLV*[Title/Abstract])) AND ((((("Vaccines"[Mesh]) OR (vaccines [Title/Abstract])) OR (vaccin*[Title/Abstract])))). Studies not identified by the above databases were included by evaluating the reference sections of relevant studies (A full list of search query used for each database is available in Supplementary material Table 2).

### Study selection and data extraction

We have included randomized clinical trials, observational studies (cross-sectional, case–control, or cohort), case series/reports, and congress and conference abstracts as a source of grey literature. The following criteria were used as our inclusion criteria; original research articles focusing on developing a HTLV-1 vaccine candidate, whether using an experimental animal model, a human-based model, or in-vitro studies investigating HTLV-1 vaccine development. The title and abstract of the studies were assessed based on the inclusion criteria after duplicate papers were removed. Finally, a thorough screening of the full texts took place. The selection was carried out independently by the two authors. Two researchers independently extracted the following data: author, year, country, type of study, number of participants (if applicable), host, vaccine type, vaccine, construct, vaccine dose, vector, route of administration, prescribed number, adjuvant, laboratory method, and study’s main findings. A third reviewer resolved disagreements.

## Results

### Characteristics of included studies

Based on our search, we retrieved 1700 citations. After removing duplicates, a total of 1250 articles were screened based on title and abstract. Furthermore, the hand-searching of other studies revealed 7 studies that met the inclusion criteria and were included after assessing their full texts. Overall, 47 articles were included in this systematic review Most of the included studies investigated the role of protein-based vaccines in developing the HTLV-1 vaccine [29–75].

### Peptide and protein vaccines

Peptid vaccines were investigated in 26 studies including 16 in-vivo, 3 in-vitro, 2 in-vivo/in-vitro and 5 in-silico studies. All in-vivo studies were animal model with animals such as mouse, rat, rabbit, and monkey. The major protein vaccine constructs assessed in these studies were comprised of Tax peptide in 15 studies [41, 44, 47–49, 51, 54–56, 63, 64, 66, 67, 73–75], Glycoprotein peptide (GP) in 9 studies [33, 35, 38, 48, 49, 58, 66, 72, 75], GAG peptide in 5 studies [47–49, 63, 66], POL peptide in 3 studies [47, 63, 66], REX and HBZ peptides each one in 1 study, respectively [47, 63].

Lairmore et al. examined inoculation of chimeric B- and T-cell epitopes of HTLV-1 env-gp46 (SP2 and SP4a) with promiscuous T-cell epitopes (from tetanus toxin and MVF protein) in mice and rabbits. They showed connecting viral peptides with promiscuous epitopes promoted specific helper T-cell responses. MVF-SP2 and SP4a-MVF constructs were efficient to overcome genetic-restricted immunity [58]. Vaccination with T and B cell epitope-based peptide constructed from the conjugation of gp46 (aa 181–210) with a branched polylysine oligomer was examined in rats and rabbits and demonstrated high HTLV-I neutralizing Abs levels. Moreover, the proliferation of T lymphocytes derived from HAM/TSP, ATLL, and asymptomatic carriers was revealed in response to construction including aa194-210 [35].

Sundaram et al. found a multivalent vaccine constructed of three HLA-A ∗ 0201 restricted CTL epitopes (Tax11–19, Tax178–186, and Tax233–241, the numbers after Tax relate to amino acids) that induced cellular immunity in HLA-A ∗ 0201 transgenic mice. Splenocyte lysis response was elicited by Tax11–19 (32%), Tax178–186 (34%), and Tax233–241 (≈6%) epitopes. Results demonstrated that Tax11–19 and Tax178–186 epitopes invoked significant CTL response and IFN-γ release in HHD mice (NSG-HLA-A2/ HHD mutant immunodeficient mice). However, Tax233–241 epitopes elicited IFN-γ release but not a significant CTL response [74]. In another study, they found vaccination chimeric constructs of B-cell epitopes derived from HTLV-1-gp21 in mice and rabbits induced neutralizing antibody response, and inhibition of syncytia formation and virus-mediated cell fusion [72]. Sundaram et al. [73] investigated epitope orientation effects in another study. CTL epitopes Tax11–19 (no. 2), Tax178–186 (no. 3), and Tax306–315 (no. 6) were used to construct 4 multiepitope vaccines with different orientations (construct 236, 632, 326, and 362). IFN-γ release and CTL response investigations demonstrated construct 236 as the most efficient, followed by construct632, against construct362 and 326. Immunity of construct 236 in mice challenging with the HTLV-1 Tax recombinant vaccinia virus showed significant viral load reduction dependent on indicated increased generation of CD8 + T-cell. splenocyte cytolytic response was shown via killing of p40-VV–infected targets by Tax (11-19)- and Tax (178–186)-stimulated splenocytes of 236 immunized mice, against no response from Tax (306–315)-stimulated splenocytes. In-vitro IFN-γ secretion was found highest in Tax (178–186)-stimulated splenocytes (670 pg/mL), followed by Tax (11–19) and Tax (306–315)- stimulated splenocytes (298 and 147 pg/mL, correspondingly) [73]. Immunogenicity investigations of another vaccine comprising three HLA-A*0201-restricted CTL epitopes derived from Tax protein (Tri-Tax) and B-cell env epitope (aa 175–218), showed antibody release against immunogen MVF–175–218 and B-cell epitope in 2/2 of squirrel monkeys. Furthermore, IFN-γ producing cell investigations resulted in three- to sevenfold increase in 2/2 immunized monkeys compared to control monkeys (0/2). Their investigations on mice challenged with HTLV-1-transformed cell lines showed proviral load reduction and strong cell-mediated response as the response [51].

Another study found a novel multi-immunodominant vaccine. The vaccine constructed of sequences of HTLV-1-Tax epitope (11–19 and 178–186) and SP2 and P21 with His-tag or mouse-Fcγ2a fusion (Tax-Env: His and Tax-Env: mFcγ2a, respectively) was examined in BALB/c mice challenging with HTLV-1-MT2 cell line by Shafifar et al. [67]. Higher IFN-γ and IL-12 secretion in “Tax-Env: mFcγ2a” and Higher IL-4 level in “Tax-Env: His” group was indicated, compared to the other group. IFN-γ, IL-12 in the Fc-fusion construct group, and IL-4 levels in the His-tag protein group were negatively correlated to proviral load. “Tax-Env: mFcγ2a” and “Tax-Env: His” demonstrated more Th1 and Th2 immune responses, respectively. They found both constructs with a 50% low proviral load of HTLV-1 and 50% complete protection in challenged mice [67].

In-vivo vaccination regimen of priming with recombinant vaccinia virus expressing whole HTLV-I envelope (gp46 and gp21) or just gp46 as a surface env protein with boosting of entire HTLV-I envelope gene, expressed in a baculovirus non-fusion vector system, demonstrated enhanced anti-env-antibody production. Neutralizing antibody level increment was shown in response to priming with recombinant vaccinia virus expressing only gp46 or with an admission of an adjuvant constructed out of mycobacterial cell wall extract [33].

### Encapsulated vaccines and vaccines with adjuvant

Eight studies investigated if adjuvants or encapsulation particles differed in the immune response to HTLV-1 vaccine constructs [30, 38, 39, 48, 49, 55, 56, 66]. PLGA (D, L-lactide-co-glycolide) encapsulation of an HTLV-1 vaccine construct demonstrated high cell-mediated and mucosal immunity [48, 49] and immunization without requiring any boosts and adjuvants, compared with free peptide vaccination [38, 39].

Frangione-Beebe et al. examined a vaccine (MVFMF2) comprising HTLV-1-gp46 (aa 175–218) linked by GPSL turn to MVF (aa 288–302). They demonstrated antibody response in mice and rabbits in the admission of N-acetyl glucosamine-3yl-acetyl-L-alanyl-D-isoglutamine (nor-MDP) adjuvant. They found promoted immunogenicity of MVFMF2 in PLGA-encapsulated form without the need for boosting and adjuvant. Anti-MVFMF2 antibodies, predominantly IgG2 (IgG2a, IgG2b) in mice, recognized HTLV- envelope protein in rabbits (*n* = 10 out of 12) and mice (*n* = 9 out of 9). Enhanced reactivity to viral antigens, viral-mediated fusion inhibition, and whole viral preparations recognition were revealed. Interestingly, the construct was not protective efficiently against cell-associated viral challenges in rabbits [38]. Kabiri et al. demonstrated the chimera multiepitope vaccination comprising HTLV-1 Tax, gp21, gp46, and gag (p19) epitopes with PLGA NPs with/without CPG oligodeoxynucleotides (ODN) elevated levels of IgG2a, mucosal IgA, IFN-γ, and IL-10 and decrease in TGF-β1 level in inoculated mice. IgG2a and IgG1 levels didn't have a significant difference in nasal and subcutaneous (SC) deliveries, but IgA level was higher in nasal administration [49]. ISCOMATRIX adjuvant admission demonstrated an increased immune response, compared to monophosphoryl lipid A adjuvant [48]. In line with the previous study [49], intranasal delivery elicited a high mucosal response compared to SC injection inducing a strong cellular-mediated response [48].

Moreover, chitosan (CHT) and trimethyl chitosan (TMC) nanoparticles demonstrated good immunoadjuvant potential in admission with a vaccine comprising env23 and env13, recombinant proteins of gp46. IgG1 and IgG total levels were demonstrated higher than antigen levels in SC injection. IgG2a titer and IgG2a/IgG1 ratio were significantly higher due to nasal delivery of env23 than SC injection. Env23 induced more potent cell-mediated immunity compared with env13 [30].

Furthermore, Schönbach et al. [66] investigated a vaccine comprised of HLA-B*3501 binding HTLV-1-peptides with the admission of C-Ser-(Lys)_4_ adjuvant. They found seven peptides derived from env-gp46, pol, gag-p19, and tax proteins invoked specific CTL responses in HLA-B*3501 transgenic mice. However, adjuvant-stimulated bulk cultures didn't show a specific CTL response.

Immunization with HLA-A*0201-restricted HTLV-1 Tax-epitope encapsulated with oligomannose-coated liposomes (OML⁄Tax) induced HTLV-1-specific CTL and IFN-γ responses, against no IFN-γ release in only peptide epitope inoculation. Moreover, dendritic cell 48 h exposure to 1 µg/ml of OML⁄Tax invoked increased CD86, MHC-I, MHC-ll, and HLA-A02 expression, in comparison [56].

Vaccination by chimeric peptide comprising HLA-A*0201-restricted HTLV1 Tax-epitope/hepatitis B virus core (HBc) particle induced HTLV-1-specific CD8 + T-cells, antigen-specific IFN-γ reaction, and anti-HBc IgG level in HLA-A*0201-transgenic mice, against only peptide inoculation. Dendritic cell 48 h-exposure to HTLV-1/HBc chimeric particle resulted in CD86, HLA-A02 and TLR4 increased expression in a dose-dependent manner [55].

Totally, studies showed nor-MDP, PLGA, ISCOMATRIX, oligomannose-coated liposomes, chitosan and trimethyl chitosan promoted immunogenicity of vaccines, in comparision with the constructs without adjuvants. However C-Ser-(Lys)_4_ adjuvant didn't show a specific CTL response.

Vaccines with anti-tumoral effects4 studies in design of in-vivo and in-vitro assessed tumor suppression/regression of their vaccines [41, 44, 53, 54]. Two in-vivo studies used mice and rats in their animal model. Tumor suppression was investigated in a study by Hanabuchi et al. [44]. They examined HTLV-I-infected T-cell line (FPM1-V1AX) inoculated rats. FPM1-V1AX inoculated rats (*n* = 2) vaccinated with a construct of Tax 180–188 and ISS-ODN adjuvant and showed tumor suppression elicited T cell immunity compared to the control group (*n* = 2). Moreover, in vivo inoculation of CTLs specific to Tax 180–188 (as a dominant recognized epitope) demonstrated tumor suppression (*n* = 2), too. Interestingly, equal antitumor effects of CD4 + and CD8 + T Cells were shown in this study as unfractionated T cells.

Furthermore, Fujisawa et al. reported 2 leukemia survival in 5 Tax-peptide vaccinated infected hu-NOG mice with restricted number and growth of infected T cells. Vaccination before infection elicited IL-12 release and Tax-specific CD8 T-cell induction [41]. Helper T lymphocytes (HTLs) reactive with Tax191–205 and Tax305–319 recognized HTLV-1 Tax–expressing T-cell lymphoma cell lines specifically, against Tax152–166 reactive HTLs, in an in-vitro study. This revealed that HTLV-1 + T-cell lymphoma cells naturally expressed these two epitopes on their surface by MHC-ll. Moreover, investigations demonstrated the natural process of these two epitopes by dendritic cells as APCs, pulsed with HTLV-1Tax + tumor lysates [54]. Kobayashi et al. [53] demonstrated an HLA-DR-bound envelope peptide similar to a fragment of human interleukin-9 receptor alpha (IL-9Ra) as an antigen associated with T-cell leukemia/lymphoma. In-vitro investigations demonstrated the induction of specific CD4 helper T lymphocytes, restricted by HLA-DR15 or HLA-DR53, in response to this synthetic peptide. These specific CD4 CTLs recognized and lysed HTLV-1 + , IL-9Ra + T cell lymphoma cells [53]. Furthermore, in-vivo assessment of MHC-I-bound HTLV-1 peptides demonstrated specific CD8 + T cell generation. IFN-γ, IL-10, perforin, MIP-1α, TNF-α, and granzyme B release from specific CD8 + T cells was shown in in-vitro investigations in the presence of MT-2 cell line [60].

Totally, these studies showed tumor suppression and leukemia survival. Main effective epitopes were found Tax 180–188 (with ISS-ODN adjuvant) [44], Tax191–205 and Tax305–319 [41] in these studies. One of the studies showed an HLA-DR-bound envelope peptide similar to a fragment of IL-9Ra as an antigen associated with T-cell leukemia/lymphoma [53].

### In-silico investigations

Investigation of designing possible Multi-Epitope Based Vaccine (MEBV) is an important progress in vaccinology as they can evoke both humoral- and cell-mediated immunity [76, 77]. Previous studies predicted engineered multiepitope-based vaccines against HTLV-1 by methodology evaluations such as B- and T-cell epitope prediction, primary, secondary, tertiary, and 3D structures modeling, antigenicity, allergenicity, and solubility prediction, homology modeling, in silico estimation and cloning, molecular dynamics stimulation, and population protection coverage calculations. These studies selected non-toxic and antigenic epitopes to construct vaccines. Antigenicity score of multiepitope vaccines in 4 studies were 0.7840 [75], 0.57 [64], 0.694 [63], and 0.4885 [47] ( at threshold = 0.5, 0.4, 0.4, and 0.4, respectively).

Tariq et al. [75] constructed a 382 amino-acid non-allergenic and non-toxic vaccine from Accessory Protein p12I, gp62, and Protein TAX-1 by selecting Cytotoxic T Lymphocytes, Helper T lymphocyte, and B cell epitopes. One of the criteria of epitopes was generating IFN-γ response. They revealed this construction had no or minimal (< 37%) homology with human proteome. In-silico estimation of the vaccine demonstrated robust IgM, IgG1, IgG2 production and cytokine and interleukin response, and positive expression of the desired protein in silico-cloning. Worldwide population coverage was revealed 95.8% with the highest coverage in India (98%), Unites States (97.14%), and Mexico (95.95%). They revealed a high binding affinity for TLR3 with a binding score of 63.8 kcal/mol and a total of 16 H-bond interactions [75]. Another vaccine predicting HTLV-1 TAX multiepitope protein constructed from CTL and B cell epitopes, Comprises 109 amino acids. All components were found non-toxic but just 3 CTL epitopes were found non-allergenic. Maximum population coverage was revealed in Mexico (90.21%), England (89.88%), and South Africa (81.56%). Strong spontaneous bindings with TLR4 and interactions of T cell epitopes with HLA-A*0201 were indicated [64]. In line with the previous study, A 808 amino-acid vaccine showed interactions with the HLA-A0201. Interactions with HLA-A0701 and HLA-A0301 receptors were demonstrated, too. The vaccine was constructed out of B‐cell, CTL, and HTL epitopes for GAG, POL, ENV, P12, P13, P30, REX, and TAX proteins. The construct was revealed probably be antigenic and non-allergenic. In silico cloning showed expression efficacy. Same as Tariq et al. [75] study Strong interaction was shown with TLR-3 [63]. Moreover, Eight B-cell and T lymphocyte epitopes were selected for 5 proteins including Gag (301–350, 217–205), Tax (142–249), Env (124–209, 354–486), Pol (155–215, 309–409), and Hbz (26–109) proteins to construct 686 amino-acid vaccines. The vaccine was investigated and found immunogenic and non-allergenic. In silico investigations indicated IgM production in initial response and IgG1, IgG2, IgG, and B cell increase in secondary response. The level of cytokines and interleukins, the population of helper and cytotoxic T lymphocytes, macrophages, and dendritic cell production were increased in response. In silico cloning, results demonstrated desired protein expression. The docking analysis demonstrated strong interaction with immune receptors, especially the HLAA*02:01 receptor [47].

Alam et al. [29] predicted 14 epitopes for a vaccine targeting Glycoprotein 62. They found strong interactions of ALQTGITLV and VPSSSTPL epitopes with HLA-A*02:03, and HLA-B*35:01, respectively. Worldwide population coverage was estimated at nearly 70%, less than Tariq et al. [75] and Raza et al. [64] study constructs. The highest coverage in West Africa (87.54%) and Europe (85.87%) was demonstrated [29]. Full characteristics of the studies are available in Table 1.

**Table 1:** Characteristics of peptide and protein vaccine studies

Author	Year	Country	Type of study	Host	Vaccine immunogen content	Vaccine dose	Route	Prescribed number	Laboratory method	Main findings
Arp, J	1993	USA	in vivo	BALB/c (Charles River), C57BL/6 (CharlesRiver) and CFW/D	envelope protein inclusion bodies (env-I.B.) in the presence or absence of an adjuvant	10 µg	IP	10 µg of env-I.B at 6 and 8 weeks of age then boosed 2 and 4 weeks later	Western blotting, radioimmunoprecipitation, peptide ELISA and a syncytium inhibition assay	Antibodies against the HTLV-I env protein in the presence or absence of an adjuvant, neutralizing Ab in admission of high doses of mycobacterial cell wall extract, enhanced Ab response to the HTLV-I envelope glycoprotein following priming with recombinant vaccinia virus (Rvv) constructs expressing either the entire native HTLV-I envelope (gp46 and gp21) or just gp46, Increased titres of neutralizing Ab following priming with the Rvv expressing gp46 only
Baba, E	1995	Japan	in vivo	Female New Zealand White rabbits and inbred female WWQdj, Fisher 433 (F433)/Qdj rats, Inbred female BN/Sea, LewisiSea rats, d ACI/Jcl rats	2 vaccines: T and B cell epitope-based peptide vaccine constructed from the conjugation of gp46 (aa 181–210) and (181–203) with a branched polylysine oligomer	500 µg (rabbit) 100 µg (rat)	IM/SC	9 New Zealand White rabbits were immunized 500 µg of pKA- or OVA-conjugated peptide (MAP181-203, MAP181- 210, 181-2030VA) emulsified with CFA on day 0 and then with IFA on days 14 and 28 5 different inbred strains of rats similarly immunized either i.m. or S.C. with 100 pg of pKA-conjugated peptide (MAP181-203, MAP181-210) emulsified with CFA on day 0 and then with IFA on days 14 and 28. Control rabbits and rats were immunized with the same amounts of CFA and IFA only	western blotting, ELISA, PCR, IF	Neutralizing Ab production in rabbits ( X4 -8 and X8-64 titers in response to MAPl 81–203 and MAPl 81 -21 0, respectively), neutralizing Abs (X40 to X320) in five different strains of rats in response to MAP1 81–210
Lairmore	1995	USA	In vivo	"Female inbred strains of mice (BALB/c, C3H/ HeJ, and C57BL/6) were obtained from Jackson Laboratories (Bar Harbor, Maine), and outbred ICR mice were obtained from Harlan Industries (Indianapolis, Ind.). Rabbits "	Chimeric and b-template peptide constructs incorporating known human T-lymphotropic virus type 1 (HTLV-1) B- and T-cell epitopes from the surface envelope protein gp46 (SP2 [aa 86 to 107] and SP4a [aa 190 to 209]) and promiscuous T-cell peptides were synthesized	100 µg in mice/ 500 µg in rabbits	SC	Booster in 3 weeks in mice/ 2 weeks in rabbits	Competitive ELISA, The radioimmunoprecipitation assay, A human osteosarcoma cell-based assay	Promiscuous T-cell epitopes, which bind to several forms of human MHC class II molecules, can be used with immunodominant peptides derived from retroviruses to produce highly immunogenic response
Schönbach	1996	Japan	in vitro and in vivo	Eight- to ten-week-old transgenic HLA-B-3501 transgenic mice of both sexes	synthetic HTLV-1 peptides mixed with the lipohexapeptide N-palmitoyl-S-[2,3-bis(palmitoyloxy)propyl]cysteinyl-seryl-lysyl-lysyl-lysyl-lysine, which is a biocompatible, Thepitopeindependent adjuvant	100 µM	intraperitoneally	NA	Peptide binding assay, Flow cytometric analysis, Western blot analysis, Cytotoxic T lymphocyte assay	CTL response in response to 11 of 37 tested HLA-B-3501 binding peptides after 3 in vitro stimulations, peptide-specific CTL induction in response to 7 peptides derived from env-gp46 (VPSPSSTPLL, VPSSSSTPL, YPSLALAPH, and YPSLALAPA), pol (QAFPQCTIL), gagp19 (YPGRVNEIL), and tax (GAFLTNVPY) proteins, Bulk CTL generation by four peptides derived from env-gp46 (SPPSTPLLY, VPSPSSTPLLY, and VPSPSSTPLL) and pol (QAFPQCTILQY) killing peptide-pulsed and recombinant vaccinia-infected target cells
Hanabuchi, S	2001	Japan	in vivo	Four-week-old female F344/N Jcl-rnu/rnu (nu/nu or athymic) rats and F344/N Jcl-rnu/ + (nu/ +) rats	synthetic oligopeptides corresponding to the Tax-epitope(180–188)	100 microg Tax 180–188 peptide alone, 10 nmol of ISS-ODN alone, 100 microg Tax 180–188 peptide mixed with 10 nmol of ISS ODN (Tax 180–188/ISS-ODN), or 100 microg Influenza A matrix 58–66 peptide mixed with 10 nmol of ISS-ODN (Influenza A matrix 58–66/ISS-ODN)	ID/IP	twice with a 2-week interval Two weeks after the last immunization, 107 freshly isolated T-cell enriched splenocytes from vaccinated rats were intraperitoneally inoculated into 4-week old nu/nu rats, which were simultaneously inoculated subcutaneously with FPM1-V1AX cells	cold inhibition assay/Cr-release assay/peptide mapping	Development prevention of FPM1-V1AX cell induced lymphomas in athymic rats in response to adoptive transfer of the Tax 180–188-specific CTL line or freshly prepared T cells from rats vaccinated with the Tax 180–188 oligopeptide in comparison with control groups, equivalent inhibitory effects on the growth of HTLV-I-infected tumors in both CD4 + and CD8 + T cells, Tax 180–188 as a dominant epitope recognized by the HTLV-I Tax-specific CTL line
Frangione-Beebe, M	2001	USA	in vivo	female New Zealand white rabbits (12 weeks)	vaccine (MVFMF2) comprising HTLV-1-gp46 (aa 175–218) linked by GPSL turn to MVF (aa 288–302)	Two rabbits: 18 mg of microspheres containing 1 mg of peptide, and 3.7 mg of microspheres containing 100 microg of adjuvant (nor-MDP) Two additional rabbits: 18 mg microspheres containing 1 mg of peptide and no adjuvant. Microspheres containing peptide or adjuvant were suspended in 1 ml of 4:1 squalene-arlacel A (Sigma) and injected intramuscularly remaining two rabbits: 1 mg of free peptide (1.3 mg/mL in PBS) and 100 mg of nor-MDP emulsied 50:50 in 4:1 squalene: arlacel A + boosting with 500 mg of peptide and 100 mg of nor-MDP at 10 weeks	IM	4 rabbits once in 12 weeks 2 rabbits in 10 and 12 weeks	ELISA, HPLC, Gun scanning electron microscope,	Sustained antibody response over a period of 5 months, without requiring a booster immunization or adjuvant in response to encapsulation of MVFMF2, elevated immune response invoked by the encapsulated peptide without requiring booster and adjuvant, Raised Ab level against both free and encapsulated MVFMF2
Frangione-Beebe, M	2000	USA	in vivo	Outbred female ICR mice female New Zealand white rabbits	MVFMF2 comprising HTLV-1-gp46 (aa 175–218) linked by GPSL turn to MVF (aa 288–302)	1 mg of peptide	IM	different for host groups	PCR, SIA, ELISA, western blot, circular dichroism (CD) spectroscopy	enhanced reactivity to viral antigens in rabbits, high titered anti-peptide antibodies in mice, immunogenic in an outbred population of both rabbits and mice when administered with adjuvant, enhanced immunogenicity when encapsulated in biodegradable microspheres without requiring of adjuvant, syncytium formation inhibition ability of anti-rabbit and anti-mouse Abs, no protection from cell-associated viral challenge in rabbits
Sundaram, R	2003	USA	in vivo	transgenic HHD mice	three HLA-A ∗ 0201 restricted CTL epitopes (Tax11–19, Tax178–186, and Tax233–241)	100 g of peptide mixed with 140 g TT3	SC	twice, 3 weeks apart	ELISPOT, Cr release assay	cellular responses to each intended epitope in vivo, high level of IFN-γ production
Sundaram, R	2004	USA	in vivo	leukocyte antigen–A-0201 monochain trans genic H-2Db -2 m double-knockout (HHD) mice	multivalent cytotoxic T-lymphocyte peptide construct derived from the Tax protein ofHTLV-1 separated by arginine spacers	100 µg of multiepitope peptide or with a mixture of 33 µg of each of the 3 individual epitopes combined with 140 µg of TT3, a pro miscuous T-helper epitope from tetanus toxoid (residues 947–967) and 100 µg of adjuvant N-acetyl-glucosamine-3-acetyl L-alanyl-D-isoglutamine (nor-MDP; Peninsula Laboratories, Belmont, CA)22 emulsified 50:50 in 4:1 squalene/Arlacel A (Sigma, St. Louis, MO)	SC	twice, 3 weeks apart	reverse-phase high-performance liquid chromatography (RP-HPLC), Cr Release Assay, interferon-gamma Release Assay, ELISA, Plaque Assay for Viral Titers	significant reduction in viral replication dependent on CD8 + T cells
Sundaram	2004	USA	in vivo	Female New Zealand outbred white rabbits, female ICR mice (8 weeks)	chimeric synthetic B-cell epitopes derived from HTLV-1-Env (gp21 and gp46) with promiscuous T-helper epitopes derived either from the tetanus toxoid (amino acids 947–967) or measles virus fusion protein (amino acids 288–302)	rabbits: initial dose (1 mg of the chimeric peptide) + booster (500 µg of peptide) / mice: initial dose (100 µg of peptide) + booster (500 µg of peptide)	s.c. in the thigh muscle in 2 rabbits, s.c in mice	rabbits [initial dose + booster injections every 3–5 weeks apart], mice [initial dose + booster at 3 and 6 weeks]	Circular dichroism spectroscopy, computer-aided analyses of protein antigenicity, Syncytia inhibition assay, Flow cytometry, Immunogenicity testing,	Neutralizing Ab against the epitopes derived from the gp21, inhibition the formation of virus-induced syncytia, peptid had secondary structure correlated well with the crystal structure data or predicted structure
Kazanji, M	2006	France	in vivo	male squirrel monkeys	three HLA-A-0201-restricted CTL epitopes derived from Tax protein (Tri-Tax) and B-cell env epitope (aa 175–218)	700 microg	IM	Two monkeys were injected twice, at 0 and 4 weeks, with the Env B-cell epitope aa 175–218 (500 mg per monkey) linked to the promiscuous T-helper cell epitope MVF (700 mg per monkey), as described pre viously (Frangione-Beebe et al., 2000). Six weeks after the first immunization, the monkeys were injected with another construct, consisting of the three Tax CTL epitopes (aa 11–19, 178–186, 306–315). Monkeys were boosted twice at weeks 9and 16 with both B- and T-cell epitopes	ELISA, PCR, western blot	high titre of Abs, high frequency of specific IFN-c-producing cells and partial protection
Kobayashi	2006	Florida	in vitro	Cell lines: EBV-LCLs, Mouse fibroblast cell lines (L-cells), HTLV-I-infected T-cell lymphoma cell lines TL-Su, TCL-Kan, HUT102, TL-Hir (HTLV-1 Tax negative), and OKM-2 T, Jurkat T-cell lymphoma cell line (HTLV-I negative), MT2,	Potential HLA-DR-restricted CD4 + T-cell epitopes of HTLV-1 Tax peptid	NA	NA	NA	Western blot analysis, ELISA, ECL detection system, chemiluminescence assay, cytokine release assay, high-performance liquid chromatography, mass spectrometry,	T-helper-cell induction in response to peptides Tax191–205 (restricted by the HLA-DR1 and DR9 alleles) and Tax305–319 (restricted by either DR15 or DQ9), Both these epitopes were naturally processed by HTLV-1 + T-cell lymphoma cells and by autologous APCs that were pulsed with HTLV-1Tax + tumor lysates. These epitopes lie proximal to known CTL epitopes, which will facilitate the development of prophylactic peptide – based vaccine capable of inducing simultaneous CTL andT-helper responses
Kozako, T	2009	Japan	in vivo	HLA-A-0201-transgenic mice	HTLV-1/hepatitis B virus core (HBc) chimeric particle incorporating the HLA-A-0201-restricted HTLV1 Tax-epitope	HTLV-1/HBc chimeric particle (20microg), or Tax11–19 peptide / HBc particle (20microg) and peptide (1microg)	intradermally	days 0 and 14 with HTLV-1/HBc chimeric particle (20 microg), or Tax11–19 peptide	ELISPOT, PCR, western blot, FCA, ELISA, enzyme-linked immunospot assay	induction of HTLV-1 Tax-specific CD8 + cells from spleen and inguinal lymph nodes after immunization, efficient induction of IFN- -producing cells, antigen-specific gamma-interferon reaction induction, increased expression of CD86, HLA-A02, TLR4 and MHC class II in dendritic cells, HTLV-1-specific CD8 + T-cells induction by peptide with HTLV-1/HBc particle from ATL patient, but not by peptide only, lysing cell presenting the peptide by HTLV-1-specific CD8 + T-cells
Kozako, T	2011	Japan	in vivo	HLA-A-0201-transgenic mice	an HTLV-1-specific CD8 + T-cell response by oligomannose-coated liposomes (OMLs) encapsulating the human leukocyte antigen (HLA)A-0201-restricted HTLV-1 Tax-epitope (OML⁄Tax)	1 microg	IM	days 0 and 14 with OML⁄Tax, Tax peptide alone or phosphate-buffered saline (PBS)	ELISPOT, FCM assay	resulted in the efficient induction of IFN-gamma-producing cells, induction of HTLV-1 Tax-specific CD8 + cells from inguinal lymph nodesafter immunization with OML/Tax, increased CD86,MHCI, HLA-A02 and MHCII levels upon exposure of dendritic cells to OML⁄Tax
Kuo, C. W	2011	Scotland	NA	NA	gp46	soluble recombinant surface glycoprotein (gp46, SU) fused to the Fc region of human IgG (sRgp46-Fc)	NA	NA	ELISA, Western blot, Syncytium interference assay, Flow cytometry,	High titer Ab responses/ Many of these mAbs recognize envelope displayed on the surface of HTLV-1–infected cells / mAbs robustly antagonize envelope-mediated membrane fusion and neutralize pseudovirus infectivity/ Potent neutralizing mAbs recognize the N-terminal receptor-binding domain / Both neutralizing and poorly neutralizing Abs strongly stimulate neutrophil-mediated cytotoxic responses to HTLV-1–infected cells
Kobayashi	2012	Florida	in vitro	Cell lines: EBV-LCLs, Mouse fibroblast cell lines (L cells), HTLV-1 infected T cell lymphoma cell lines, TL-Su, TCL-Kan,OKM-2 T, Hut102, TL-Hir, Jurkat T cell lymphoma cell line, prostate tumor cell line PC3, and PBMCs	HLA-DR-bound peptide from the IL-9 receptor alpha of HTLV-1-transformed T cells	NA	NA	NA	Purification of HLA-DR molecules, Preparation of bound peptides, Binding assay, Western blot analyses, Cell-mediated cytotoxicity assays,	antigen-specific CD4 helper T lymphocytes generation (in vitro) restricted by HLA-DR15 or HLA-DR53 molecules with recognizing and killing ability of HTLV-1 + , IL-9Ra + T cell lymphoma cells
Fujisawa	2015	Japan	in vivo	HTLV-1-infected humanized mouse model (hu-NOG) mouce	Mixture of twelve overlapping peptides of 40–42 amino acids long encompassing whole Tax protein	NA	subcutaneously	vaccine was inoculated subcutaneously three times weekly to hu-NOG mouse and then -irradiated HTLV-1 producing Jurkat cells were intraperitoneally injected to infect HTLV-1	NA	leukemia suppression, retardation of the out growth of human lymphocytes in response to Tax-immunization after HTLV-1 infection, survival of two out of five mice with alimited number of infected T-cells, IL-12 induction and enhanced expression of Tax-specific CD8 T-cell in immunized mice before infection
Amirnasr, M	2016	Iran	in vivo	male BALB/c mice	env23 (162–209) and env13 (125–209) recombinant proteins	7.5 µg antigen	nasal/SC	3 immunizations (7.5 µg antigen) were performed with 2 weeks intervals	ELISA, PCR	higher serum IgG1 and IgG total levels compared to antigen solution, higher IgG2a levels and IgG2a/IgG1 ratio in nasal delivery compared with subcutaneous administration (*P* < 0.001), higher cellular immune responses in response to env23 antigen, compared with env13
Kabiri, M	2018	Iran	in vivo	BALB/c male mice	chimeric peptide vaccine including Tax, gp21, gp46, and gag immunodominant epitopes of human T-cell lymphotropic virus type 1 (HTLV-1)	10 microg	nasal/SC	three times at two weeks intervals	ELISA, PCR	increased Ab titers containing IgG2a, mucosal IgA, as well as IFN-γ and IL-10 cytokines and decreased TGF-β1 level in response to mixture of IMX and chimera, potent mucosal sIgA titers in intranasal delivery compared to subcutaneous root, cell-mediated responses, as evident by higher IgG2a and IFN-γ, as well as suppressed TGF-β1 level in SC or nasal delivery
Kabiri, M	2018	Iran	in vivo	BALB/c male mice	chimeric peptide vaccine including Tax (aa 11–19 and aa 178–186), gp21 (aa 370–400), gp46 (aa 165–306), and p19 (aa 105–124) immunodominant epitopes of human T-cell lymphotropic virus type 1 (HTLV-1)	10 microg	nasal/SC	three times at two weeks intervals	ELISA, PCR, western blot	elevated titers of IgG1, IgG2a, and sIgA antibodies, as well as IL-10, and IFN-γ cytokines and decreased TGF-β1 level, promoted cellular and mucosal responses in co-delivery of chimera and CpG ODN in PLGA
Mulherkar	2018	USA	in vivo and in vitro	Six to eight-week old female HLA-A2 transgenic mice / Cell lines for in vitro investigations: HepG2, hepatoma cells, MT2, HTLV-1 virion expressing cells, and T2, TAP deficient lymphoblasts	MHC-I-bound HTLV-1 peptides	NA	interadermal near the base of the tail and subcutaneous on the flank	Three injections: initial inoculation (consisted of a mixture of pooled free peptide in PBS plus Montanide ISA 51 (Seppic, Paris, France) (50:50 emulsion), PBS alone, or two independent, individual free peptide in PBS plus Montanide ISA 51 (50:50 emulsion)) + repeated two more times at 10-day intervals)	Degranulation assay, CD8 + T-cell killing assay, Mass spectrometry analysis, Flow cytometry analysis, ELISpot assays, MagPix cytokine detection,	confirmation of six novel MHC-I restricted epitopes capable of binding HLA-A2 and HLA-A24 alleles, generation of CD8 + T cells specific for each of these peptides, generation of epitope-specific CD8 + T cells secreted IFN-γ, granzyme B, MIP-1α, TNF-α, perforin and IL-10 in the presence of MT-2 cell line in vitro, cytotoxic response through surface expression of CD107 on CD8 + T cells when cultured with MT-2 cells, significant antiviral activity of CD8 + T cells specific against all identified peptides, In vivo generation of CD8 + T cells similarly demonstrated immunogenicity on ELISpot, CD107 degranulation assay, and MagPix MILLIPLEX analysis
Pandey	2019	India	in silico	NA	vaccine by the assimilation of B‐cell, CTL, and HTL epitopes for GAG, POL, ENV, P12, P13, P30, REX, and TAX proteins	NA	NA	NA	B‐cell, Helper T‐cell (HTL), and Cytotoxic T‐cell (CTL) epitope prediction, Tertiary structure prediction, Molecular docking, in silico cloning	interactions with the HLA-A0201, HLA-A0701 and HLA-A0301 receptors, Strong interaction with TLR-3
Alam	2020	Bangladesh?	in silico	NA	prediction of 14 epitopes for targeting Glycoprotein 62	NA	NA	NA	Variability Analysis of GP62 of HTLV-1, Population Protection Coverage (PPC) Calculation, HLA-Epitope Binding Prediction, Molecular Dynamics Simulation, Prediction ofB-Cell Epitope,	ALQTGITLV and VPSSSTPL epitopes interaction with three MHC alleles ( including HLA-A-02:03, and HLA-B-35:01, respectively), 70% summative population protection coverage
Jahantigh	2021	Iran	in silico	NA	eight-epitopes-rich domain, including overlapping epitopes detected on both B and T cells constructed of Gag, Env, Pol, Hbz, and Tax proteins	NA	NA	NA	antigen prediction, Mapping, 3D Structure modeling, Homology modeling, Antigenicity and allergenicity and solubility and other physicochemical parameters evaluation, structure prediction, In silico cloning, Immune simulation, peptide–allele docking	interaction of the epitope and the designed protein with immune receptors(in silico docking), strong interaction of O2 epitope and D8 protein with immune receptors especially the HLAA 02:01 receptor, stability of the interactions for 100 ns(molecular dynamic), root mean square deviation, radius of gyration, hydrogen bonds, and solvent-accessible surface area were calculated for the 100 ns, humoral and cell-mediated immune responses elicited
Raza	2021	Bangladesh	in silico	NA	predicting HTLV-1 TAX multiepitope protein constructed from CTL and B cell epitopes	NA	NA	NA	Primary, secondary, tertiary and 3-D structure analysis, B- and T-cell epitope prediction, molecular docking analysis, Disulfide engineering, in silico cloning,	most antigenic score of 0.57, strong T cell epitopes interaction with HLA-A-0201, high binding affinity of the vaccine construct for TLR4 (in molecular docking study), most antigenic and immunogenic epitopes in in-silico investigation: B cell epitopes (KEADDNDHEPQISPGGLEPPSEKHFR and DGTPMISGPCPKDGQPS spanning from 324–349 and 252–268 respectively); T cell epitopes (LLFGYPVYV, ITWPLLPHV and GLLPFHSTL ranging from 11–19, 163–171 and 233–241)
Tariq	2021	Pakistan	in silico	NA	9 Cytotoxic T Lymphocytes, 6 Helper T Lymphocytes and 5 Linear B Lymphocytes epitopes, joint through linkers and adjuvant	NA	NA	NA	Conservation analysis and selection of predicted epitopes, Epitope modeling and molecular docking, homology analysis, Disulphide engineering, In-silico estimation and cloning,	strong binding affinity with their corresponding Human Leukocyte Antigen alleles, 95.8% coverage of the world’s population, highly antigenic properties while being non-toxic, soluble, non-allergenic, and stable in nature, enhanced stability via disulphide engineering, strong association between vaccine construct and human pathogenic immune receptor TLR3 (in Molecular docking analysis and Molecular Dynamics (MD)), rapid antigen clearance and higher levels of cell-mediated immunity in response to repeated-exposure and immune simulations, respectively
shafifar	2022	Iran	in vivo and in vitro	male 6 to 8 weeks pathogen-free BALB/c mice	Fc-fusion multi-immunodominant recombinant protein (Tax-Env: mFcγ2a and Tax-Env: His)	1) Six mice received 50 μg of tTax-tEnv:mFcγ2a in 100 μL PBS + 100 μL of DDA adjuvant /2) Four mice, 50 μg of tTax-tEnv:His in 100 μL of PBS + 100 μL of DDA adjuvant / 3) Five mice, 200 μL of PBS (negative control)	intraperitoneal	T200 μL/mouse thrice at two-week intervals (0, 14th, and 28th days)	SDS-PAGE, Western blot, real time PCR	significant increase in IFN-γ and IL-12 release in response to Tax-Env: mFcγ2a compared to Tax-Env: His, 50% low proviral load of HTLV-1 and 50% complete protection in challenged mice, more Th1 immune responses in response to "Tax-Env: mFcγ2a”, more Th2 immune responses in response to “Tax-Env: His”

### DNA vaccines

All DNA plasmid vaccines in this literature review were in-vivo animal studies (Table 2). Armand et al., 2000 [32] compared two plasmid vaccines containing the whole HTLV-I envelope gene under the control of the CMV promoter (CMVenv or CMVenvLTR) and human desmin muscle-specific promoter (DesEnv). DesEnv inoculation demonstrated sooner and higher anti-envelope antibody response, compared with CMVenv/LTR vaccination. Consistent with this study, Grange et al. [42] showed single CMVenv or CMVenvLTR could not elicit generating detectable antibody levels. However, boosting with gp62 baculovirus recombinant protein demonstrated detectable HTLV-I-env antibody levels. Kazanji et al. [50] found different results for two immunization regimens. The first regimen was the inoculation of recombinant HTLV-I-env adenovirus or naked DNA plasmid and boosting with Ad5 containing the gp46 gene or with baculovirus-derived recombinant gp46 in WKY rats. No detectable antibodies were found after this regimen compared to the second regimen, priming and boosting with HTLV-I-env gene recombinant vaccinia virus in F-344 rats. CTL response in response to the first regimen was found higher than natural in response to the first regimen, but to the same extent in rats primed with either Ad5-HTLV-I-env or the naked plasmid. There were no changes with boosting. Ohashi et al. [62] found vaccination of F344/N rats with plasmids containing wild-type Tax cDNA driven by the β-actin promoter induced Tax-specific CTLs. But in contrast, no antibody levels were detected. Nakamura et al. [61] demonstrated vaccination of 4 cynomolgus monkeys with the env gene, produced by the Escherichia coli system, elicited a specific Anti-HTLV-I-env humoral response in 2 monkeys. They showed immunity against HTLV-1 producing cell line infection in these 2 monkeys against 2 others which inoculated with low doses of vaccine construct.

**Table 2:** Characteristics of DNA vaccine studies

Author	Year	Country	Type of study	Host	Vaccine immunogen content	Vaccine dose	Route	Prescribed number	Laboratory method	Main findings
Nakamura, Hideo	1987	Japan	in vivo	Cynomolgus monkeys (Macaca fascicularis)	env gene products of HTLV-I produced in E.coli	different (100 or 150 microg)	ID/IV	different for each group	SDS–polyacrylamide gel electrophoresis, Western blot, IFA	Ab against HTLV-I gp68 and gp46, strong inhibition of syncytium formation, humoral immunity
Grange, M. P	1997	France	in vivo	6 to 8 week old male BALB/c mice	complete HTLV-I envelope	10 microg for protein	IM (vector)/IP(protein)	[different protocls were used] Mice were immunized IP with 10 µg of gp62 Baculovirus recombinant protein in complete Freund's adjuvant followed by three boosting doses of 10 microg of recombinant protein in incomplete Freund's adjuvant at 2-week intervals. Two mice of each DNA-primed group were immunized with protein at 14 weeks post-DNA inoculation. For comparative studies, two naive mice were immunized with protein with the same protocol	ELISA, IFA, syncytium formation assay, CTLL assay	high antibody response in response to protein boosts in mice primed with DNA expressing HTLV-I envelop proteins, high neutralizing antibody titers, memory B-cell clone stimulation via single inoculation of DNA expressing HTLV-I env gene, specific cellular helper cell response in mice
Kazanji, M	1997	France	in vivo and in vitro	WKY and Fischer F-344 rats	The complete human T-cell leukemia virus type I (HTLV-I) env gene was inserted into an expression cassette containing the adenovirus 5 major late promoter (Ad5-MLP). (Recombinant Ad5-HTLV-I-env)	Fischer F-344 rats: 107 PFU of WR-SFB5 env or control HA-WR/ WKY rats: 200 µl PBS containing 109 PFU of Ad5-HTLV-I-env (or Ad5-HTLV-I-gp46 for boosting) or 100 µg of the naked DNA expression vector pMLP-HTLV-I-env. Booster injections with baculovirus-derived recombinant gp46 (1 µg) were delivered subcutaneously together with 50 µg of saponin as adjuvant	IM/intradermally	different for host groups	IFA, western blot, PCR, SIA, CTL assay	WKY rats: No detectable Ab against HTLV-I, recovery of HTLV-I-specific cytotoxic T lymphocytes in all immunized groups but not from controls, Fischer F-344 rats: Ab against the HTLV-I env gp21 and gp46 (non-neutralizing), partial protection in both immunization regimens after challenge with human HTLV-I-producing cells (MT-2)
Ohashi, T	2000	Japan	NA	Female F344/N Jcl-rnu/rnu (nu/nu) rats and F344/N Jcl-rnu/1 (nu/1) rats	Tax-coding DNA	10 μg	The Helios Gene Gun system	twice, with a 1-week interval	Cr-release cytotoxicity assay, SDS-PAGE,	Tax-specific CTL induction, CTLs ability to lyse HTLV-1 infected syngeneic T cells in vitro, in vivo growth inhibition of HTLV-1-transformed tumor, efficient anti-tumor immunity induction
Armand, M. A	2000	France	in vivo	female BALB/c mice	two types of plasmids for DNA: 1) coding DNA of the complete env gene of HTLV-I under the control of the CMV promoter with (CMVenvLTR) or without (CMVenv) the tax/rex genes, 2) coding DNA of the complete env gene of HTLV-I under the control of the human desmin muscle specific promoter (DesEnv)	100 microg	IM	3 immunizations were performed with 2 weeks intervals	PCR, Flow-cytometry, ELISA and neutralization assays	detectable and neutralizing humoral response, higher humoral response with better neutralization properties in response to the DesEnv construct compared to CMVenvLTR or CMVenv plasmids

### Dendritic cell-based vaccine

Dendritic cell-based constructs were suggested as therapeutic vaccines that induced specific CD8-T cells [31, 65, 70] (Table 3). Sagar et al. [65] suggested Tax (11–19) epitope as a potential candidate for the DC-based anti-HTLV-1 vaccine. They reported induction of antigen-specific CD8 T cell in response to Tax (11–19) epitope in presence of dendritic cells (DCs), against no response in DC depletion in an in-vivo HLA-A2/DTR hybrid mice study. They also indicated Freund’s adjuvant admission decreased TGF-β and potentiated CD8 T lymphocyte response [65]. A human clinical trial of 3 previously treated ATL patients investigated the therapeutic efficacy of Tax peptide-pulsed dendritic cells with SC injection. Specific CTL responses were elevated. Partial remission was reported in 2 patients in the first 2 months. Complete remission was seen in one of these patients. Remission status maintained 24 and 19 months after injection without requiring any additional chemotherapy. Inconsistently, the third patient showed developed progressive disease slowly, but additional chemotherapy was not needed for 14 months. The first patient showed diarrhea, fever, and dermatitis and the second and third patients showed only fever and dermatitis as not severe adverse effects [70]. Proviral load reduction and Tax-specific CD8 + T cells induction was demonstrated in response to Tax-specific CTL epitope–pulsed DC immunotherapy in infected mice by Ando et al. [31].

**Table 3:** Characteristics of dendritic-cell-based vaccine studies

Author	Year	Country	Type of study	Host	Vaccine immunogen content	Vaccine dose	Route	Prescribed number	Laboratory method	Main findings
Sagar, Divya	2014	USA	in vivo	Transgenic hybrid mice generated from an intercross between HLA-A2.1 and DTR transgenic mice / HLA-A2.1 transgenic mice / DTR transgenic mice the last two types were used to produce the hybrid mice	Tax(11–19) epitope	100 μg	ID/SC	once	PCR/ELISA/MILLIPLEX magnetic bead assay	reduced proliferation of CD8 + splenocytes from Tax 11–19 immunized DC depleted mice, higher frequency of Tax 11- 19-specific cells with adjuvant usage, Tax 11–19 epitope as a potential candidate for a DC-based anti-HTLV-1 vaccine
Suehiro, Youko	2015	Japan	human	human	autologous dendritic cells (DCs) pulsed with Tax peptides corresponding to the CTL epitopes	$5\times10^6$	SC	three times at 2-week intervals	PCR	Tax specific CTL response, partial remission in 1 patient, complete remission in 1 patient, maintaining remission status without any additional chemotherapy, progressive disease in 1 patient,
Ando, S	2017	Japan	in vivo	Three- to six-week-old female rats (F344/N Jcl-rnu/ +)	HTLV-1 Tax(180–188)-specific CTL epitope-pulsed dendritic cell therapy	$1\times10^6$ cells	SC	once a week for 3 wk into rats	PCR, ELISA, Flow cytometry,	monocyte-derived DCs capacity to stimulate CMV-specific autologous CTLs in vitro, peptide-pulsed DC immunotherapy will be useful to induce functional HTLV-1–specific CTLs and decrease PVL in infected individuals with high PVL and impaired HTLV-1–specific CTL responses, therby reducing the risk of the development of ATL

### Recombinant vaccinia virus

The use of vaccinia virus as a tool for developing vaccines is evident in literature [78]. Previous studies supported the use of this technique to develop vaccines against influenza virus [79], parainfluenza virus [80], and human immunodeficiency virus type 1 (HIV-l) [81]. Regarding HTLV-1, our search identified 8 studies which used vaccinia virus to develop HTLV-1 vaacine [34, 36, 43, 45, 52, 68, 69, 71, 82]. Except three [52, 71, 82], all studies were conducted before 2000 [34, 36, 43, 45, 68, 69]. One of the studies were an in-vitro study performed by Arp et al. [34] and was aimed to express HTLV-1 gp46 envelope protein in a vaccinia virus. All remaining studies were animal studies performed on rabbits [43, 68, 69], mice [36, 71], and monkeys [45, 71]. The most recent study by Sugata et al. showed using a recombinant vaccinia virus (rVV) vaccine expressing HTLV-1 basic leucine zipper (bZIP) factor (HBZ) or Tax induced specific T-cell responses to HBZ and Tax in HTLV-1–infected monkeys [71]. They proposed HBZ157-176 as a candidate peptide for future vaccine developments for this virus while high level of HBZ-specific CTLs were noticeable after inoculation. Two reports by Shida et al. were mainly focused on finding a new site in vaccinia virus for insertion of foreign genes such as HTLV-1 envelope gene [69] and proposing LC16mO as a potential vector [68]. Use of WR-SFB5env constructed vaccine was accompanied by a noticeable immune response. Antibody titers were still recognizable after 2.6 years following the infection [45]. However, these results were in contrast with those published by Hakoda et al. [43]. Compared with controls, rabbits which received WR-SFB5env constructed vaccine were became infected again after receiving an infected HTLV-1 blood (3 out of 3 in control and 2 out of 3 in WR-SFB5env group). In the study by Ford et al. three different construction were developed for assessing the efficacy of rVV vaccine [36]. Depending on the sort of animals used for experiment, vaccination outcomes varied greatly [36]. A combination vaccine therapy using vaccinia virus-derived NYVAC vaccine and a DNA based vaccine has been investigated previously [52]. Administration of a DNA immunogen CMV-env-LTR before immunization with HTLV-1 gag/env NYVAC vaccine showed a full protection among all three inoculated monkeys. Therefore, they suggested live recombinant vector-based vaccine as a potential booster candidate following separate DNA vaccination, as the results showed both humoral and cell-mediated immunity were maintained at its highest level (Table 4).

**Table 4:** Characteristics of recombinant-vaccina-virus vaccine studies

Author	Year	Country	Type of study	Host	Vaccine immunogen content	Vaccine dose	Route	Prescribed number	Laboratory method	Main findings
Shida, H	1987	Japan	in vivo	rabbits	The envelope gene of HTLV-I in the vaccinia virus hemagglutinin (HA) gene	NA	ID	once	IFA	HA gene is a useful site to accept and express foreign genes/ A single inoculation of the recombinant virus-induced antibodies to the env proteins of HTLV-I in rabbits and had a protective effect against HTLV-I infection
Shida, H	1988	Japan	in vivo	Japan albino rabbits, each weighing 1.8 to 2.3 kg and 5-week-old male DDY mice	HTLV-1 envelope gene	NA	IP/IC	once	IFA	LC16mO is a good candidate as a vector for vaccination
Ford, C. M	1992	USA	in vivo	Balb/c, A/J, and C57BU6 strains of mice	RVV El expressed the native HTLV-I envelope proteins gp46 (surface protein) and gp21 (transmembrane protein) RVV E2 expressed the envelope precursor with the proteolytic cleavage site deleted RVV E3 construct expressed only the external surface glycoprotein (gp46)	NA	intraperitoneal	NA	Southern blot, Immunofluorescence assays, Radioimmunoprecipitation assays, ELISA, Western blot assays	Balb/c mice responded poorly to immunization with all of the three RVV constructs. C57BU6 mice produced neutralizing antibodies in response to immunization with all three constructs, whereas A/J mice developed neutralizing antibodies only when immunized with the RVV El s construct. The results indicate that the humoral immune responses depend on the form of HTLV-I envelope proteins expressed by each RVV
Hakoda, E	1995	Japan	in vivo	Japanese white rabbits	env gene in the hemagglutinin locus, WR-SFBSenv	$1\times10^{8}$ plaque-forming units of recombinant or control virus at 3 sites on the back	intradermally	one time in 3 sites in the back of tabbits	PCR, western bloth, ELISA, plaque-reduction assay	Incapable of inducing neutralizing antibodies
Arp, J	1996	USA	in vitro	baculovirus non-fusion vector system	gp46	NA	NA	NA	PCR, ELISA, western blot, y immunofluorescence assays	Maintenance of highly conserved conformational epitopes in the recombinant HTLV-1 envelope protein structure
Ibuki, K	1997	Japan	in vivo	cynomolgus monkeys (Macaca fascicularis)	HTLV-I envelope (Env) gp46	Two monkeys: 3.10 p.f.u. of WR-SFB5env three monkeys: $3.10^{8}$ p.f.u. of HA − VV	ID	1	western blot, PCR, particle agglutination, IFA	Neither HTLV-I antigen nor HTLV-I proviruses were detected/ Single immunization with WR-SFB5env elicited long-lived anti-Env antibodies as well as Env-specific CTL activity/ Gp46 expression alone was sufficient for protection
Kazanji, M	2001	France	in vivo	male squirrel monkeys	env/gag	In the initial protocol, three monkeys $10^{8}$ PFU of NYVAC / Six months after the last administration of NYVAC-env, two of the three vaccinated monkeys were boosted with 500 mg of the naked DNA immunogen CMV-env-LTR, The third monkey and the control were injected with a naked DNA vector containing the b-galactosidase gene (CMV-bgal) In the second immunization protocol, three monkeys 500 mg of the DNA immunogen CMV-env-LTR and the control monkey was injected with the CMV-bgal vector. Six months later,the three vaccinated monkeys received a series of three booster injections, separated by 1-month intervals, of 108 PFU of the NYVAC-based candidate vaccine containing the HTLV-1 env and gag genes. The control monkey received 108 PFU of NYVAC-RG at the same times	IM	protocol A: (3 monkeys) 0, 1, and 3 months (108 PFU of NYVAC containing the HTLV-1 env gene) Six months after the last administration of NYVAC-env, two of the three vaccinated monkeys were boosted with 500 mg of the naked DNA immunogen CMV-env-LTR intramuscularly into the tibialis anterior muscle protocol B: (3 monkeys) 500 mg of the DNA immunogen CMV-env-LTR. Six months later, received a series of three booster injections, separated by 1-month intervals, of 108 PFU of the NYVAC-based candidate vaccine contain ing the HTLV-1 env and gag genes	ELISA, PCR, western blot	protocol A: With the first immunization protocol, no anti-bodies against HTLV-1 HTLV-1 Env gp46 was stimulated in all of the three immu nized monkeys and a lesser response was stimulated in the control monkey protocol B: did not induce detectable levels of antibodies against HTLV-1 In the lymphocyte proliferation test performed 1 month after boosting, high-level, specific responses were detected in the three immunized animals against both recombinant Env gp46 protein and Gag peptides but not in the control monkey
Sugata, Kenji	2015	Japan	in vivo	Ly5.1 C57BL/6 mice, rhesus monkeys	HTLV-1 basic leucine zipper (bZIP) factor (HBZ) or Tax	Each animal received a dose of 107 plaque forming units of rVV in 10 mL of viral suspension	skin sacrifation	In mice, 4 weeks after the first vaccination, 5 booster vaccinations were administered every 3 weeks In monkeys, booster vaccinations were repeated every 4 weeks. PBMCs from monkeys were obtained every 2 weeks	ELISPOT, immunoblotting	Increased survival of the lymphoma cell–inoculated mice/ Induction of specific T-cell responses to HBZ and Tax in HTLV-1–infected rhesus monkeys/ A candidate peptide (HBZ157-176) for vaccine development was identified/ Dendritic cells pulsed with this peptide could generate HBZ-specific CTLs from human CD81 T cells

### Other proposed vaccines

Kuo et al. have used a recombinant surface glycoprotein (gp46) attached to the Fc region of human IgG (sRgp46-Fc), which lead to a significant rise in the antibody (Ab) response [57]. Furthermore, the results of this recombinant glycoprotein-based vaccine revealed that the majority of these antibodies recognized HTLV-1-infected cells and inhibited virus fusion to the cells. The robust antagonizing activity of Abs was mostly seen in the N-terminal region of gp46. As an important observation, strong neutrophil response to HTLV-1 infected cells were also reported. The use of attenuated poxvirus vaccine vectors (ALVAC and NYVAC) for immunization of New Zealand White rabbits were described by Franchini et al. [37]. Gp63 was the HTLV-1 envelope protein used in the vaccine construction. Two immunization was done within 1 month, and the results showed full protected rabbits after 5 months of last inoculation.

The use of ATLL patients own peripheral blood mononuclear cells (PBMC) were also suggested to have an immunogen activity against the virus through activating Tax- specific CTLs [46]. Expressing Tax antigen, IL-12, and other stimulatory molecules in a cultured environment with the presence of both HTLV-1 infected cells and the patients' PBMC leads to CD8 + Tax-specific CTL responses. These findings could recommend a future vaccine candidate through the use of these stimulated PBMCs.

In a study by Fujii et al., an anti gp46 antibody was used for a possible induction of passive immunization in two pregnant rats [40]. In their in-vitro investigation, using 5 µg/mL monoclonal antibody of rat origin (LAT-27) completely blocked HTLV-1 infection. Moreover, newborn rats of mothers with pre-infused mentioned antibodies showed complete resistance against HTLV-1 (Table 5).

**Table 5:** Characteristics of other proposed vaccine studies

Author	Year	Country	Type of study	Host	Vaccine immunogen content	Vaccine dose	Route	Prescribed number	Laboratory method	Main findings
Franchini, G	1995	USA	in vivo	New Zealand White rabbits	attenuated poxvirus vaccine vectors (ALVAC and NYVAC) with the use of Gp63	$10^7$ plaque-forming units [PFU]	IM	2 immunizations were performed with 1 month interval	IFA, PCR, syncytia inhibition assay	The results indicated that two inoculations of the ALVAC-based HTLV-1 env vaccine candidate protected animals against viral challenge 5 months following the last immunization
Kuo, C. W	2011	Scotland	NA	NA	recombinant surface glycoprotein (gp46) attached to the Fc region of human IgG (sRgp46-Fc	soluble recombinant surface glycoprotein (gp46, SU) fused to the Fc region of human IgG (sRgp46-Fc)	NA	NA	ELISA, Western blot, Syncytium interference assay, Flow cytometry,	High titer Ab responses/ Many of these mAbs recognize envelope displayed on the surface of HTLV-1–infected cells / mAbs robustly antagonize envelope-mediated membrane fusion and neutralize pseudovirus infectivity/ Potent neutralizing mAbs recognize the N-terminal receptor-binding domain / Both neutralizing and poorly neutralizing Abs strongly stimulate neutrophil-mediated cytotoxic responses to HTLV-1–infected cells
Fujii, H	2016	Japan	in vivo	Strains of SD rats	anti gp46 (191–196)antibody	25 mg/head of either LAT-27 or isotype control mAb two times	IP	two times on –7 d and –2 d of delivery	ELISA, qPCR, Flow Cytometry, SIA	When humanized immunodeficient mice were pre-infused intravenously with humanized LAT-27 (hu-LAT-27), all the mice completely resisted HTLV-I infection. These results indicate that hu-LAT-27 may have a potential for passive immunization against both horizontal and mother-to-child vertical infection with HTLV-I
Ishizawa, M	2021	Japan	in vitro	NA	Mitomycin C-treated HLA-A2-negative HTLV-1-infected T-cell lines or short-term cultured peripheral blood mononuclear cells (PBMC))	NA	NA	NA	ELISA, PCR, CTL assay	Short-term cultured autologous PBMC from ATL patients could potentially serve as a vaccine to evoke Tax-specific CTL responses
Lucchese	2021	Germany	in vitro	NA	mRNA and Peptide-Based Vaccines	NA	NA	NA	NA	An epitope platform for HTLV-1 vaccine have been presented to reduce post-vaccination adverse events, cross-reactivity with human antigens

## Discussion

**Fig. 1:**
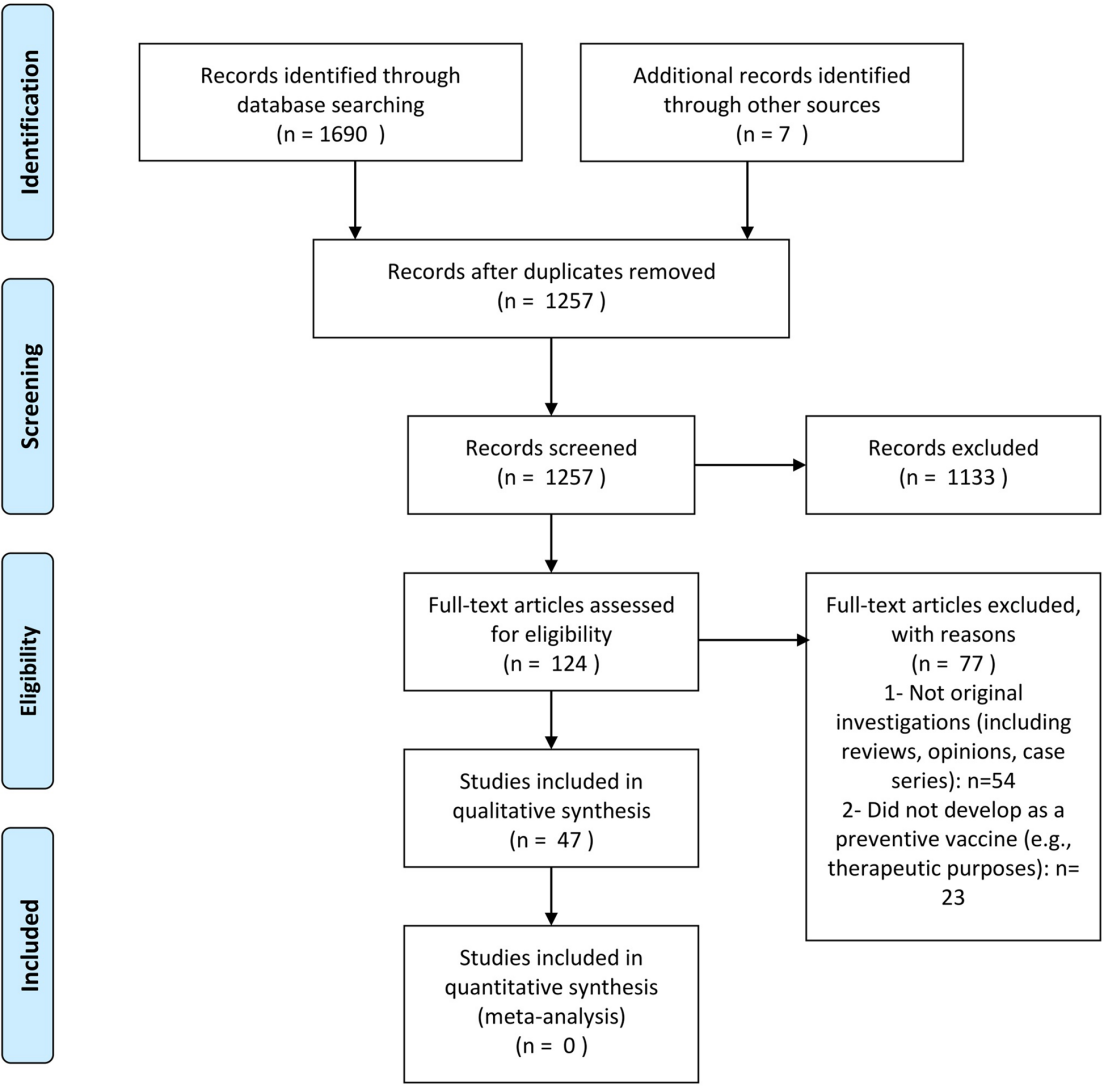
Database search and selection

This is, to the best of our knowledge, the most comprehensive systematic review that thoroughly reviewed the available evidence regarding multiple efforts to create a well-developed vaccine against HTLV-1. In this paper, we reviewed the findings from 47 studies which used several different methods to design the aforementioned vaccine, including peptide/protein, DNA-based, dendritic-cells-based, and recombinant vaccinia virus. Most of the included studies were peptide or protein based experimental models, which mostly used Tax, Glycoprotein (GP), GAG, POL, REX, and HBZ as their peptides to develop the vaccine. Dendritic cell-based studies were more recently published and achieved their immunization through an activated CD-8 response. The first attempts to create an HTLV-1 vaccination relied on recombinant vaccinia virus and most results sound positive and efficacious, albeit there hasn't been much focus regarding this type of vaccine lately. Most of the studies were experimental studies performed on animal models, although few investigations were done on humans. CMV, vaccinia, baculovirus, hepatitis B, measles, pox, E. coli, and adenovirus were among the most commonly used vectors in the studies (Fig. 1).

In addition to our predefined database search, we also systematically searched the Cochrane library (CENTRAL) to gather recent progression and future perspectives regarding the evaluation of the HTLV-1 vaccine in clinical trials. The most recent randomized controlled study protocol by Suehiro et al. is registered in the Japan Registry of Clinical Trials and aims to evaluate the effectiveness of autologous dendritic cell vaccine therapy in adult T-Cell Leukemia/Lymphoma (ATLL) patients. The main population were pre-treated ATLL patients and positive for any of HLA-A*0201, *2402, *1101, or *0207. The three times with a two-week interval at a dose of 5.0 × 106 cells vaccine will be subcutaneously injected and patients’ progression-free survival, the vaccine safety and effectiveness will be reported. Moreover, another protocol submitted by Suehiro et al. was aimed at studying the effectiveness of autologous dendritic cell vaccine pulsed with Tax peptides in ATLL patients. However, this study was terminated. A few other protocols were available, but none of them reported their results. Therefore, the authors of this systematic review urge further investigation into the potential use of these suggested vaccines to prevent and treat HTLV-1 infection in humans based on their efficacy in animal models.

Previous reviews are also available in the literature regarding developing an efficacious HTLV-1 vaccine [83–86]. The most recent study by Santana et al. systematically reviewed the last 35 years efforts for developing HTLV-1 vaccine [83]. In their study, 25 articles were included, out of which 19 were peptide based, and 6 were viral vector-based vaccines. The authors focused on including only the articles with strong evidence and excluded those articles which discussed new strategies to develop HTLV-1 vaccine. In our article, we also included recent advances in developing HTLV-1 vaccine which includes but not limited to dendritic cell-based vaccines, recombinant vaccines, and use of ATLL patients own PBMCs. Furthermore, we discussed in-vitro investigation in addition to animal and human models.

Our study has several limitations. First, due to the heterogeneous results and methodology of each study, meta-analysis was not carried out and the result section was presented in narrative form. Second, because the number of studies evaluating the effects of the proposed HTLV-1 vaccine in humans was insufficient, the applicability of the efficacy of the experimental animal models is unknown. Finally, little information was available regarding the comparison of the effects of the different proposed vaccine types to each other.

In conclusion, this systematic review summarized recent assessments of HTLV-1 vaccine candidates. There are numerous constructs with potential immunogenicity investigated in in-silico, in-vitro, and in vivo studies. Cell-mediated immunity, tumor suppression, leukemia regression, and humoral response with antibody secretion were reported in reviewed studies. HTLV-1-Tax epitopes (especially 11–19 and 178–186) and gp46 and gp21 were the most used epitopes in different immunogen vaccines. Some dendritic-cell-based and Tax epitope (180–188)-based vaccines showed reducing risk of the development of ATL in vivo. Although human clinical trials for HTLV-1 vaccines remain rare yet, a 3-individual-human trial showed the therapeutic efficacy of autologous dendritic cells for ATL patients. Recent in silico studies predicted the highest immunogenic T- and B-cell epitopes for efficient HTLV-1 vaccine. Further wet lab and in vitro investigations are required to authorize their vaccines. Elevated cell immunity appeared to be associated with Tax-specific CTL responses and protection from illness. Encapsulation of the vaccine with some nanoparticles (such as PLGA) showed the same immunity without the need for adjuvants or boosting. This study will address the essential need for a potential HTLV-1 vaccine to prevent and or treat ATLL and other HTLV-1 immune-related disorders. It is difficult to determine which approach is the most promising for developing an HTLV-1 vaccine, as each approach has its own advantages and disadvantages. Additionally, each approach may work differently in different populations and may have different safety and efficacy profiles. However, some of the approaches that have shown promising results in preclinical studies include the use of peptide vaccines, virus-like particle (VLP) and adenoviral vector vaccines encoding HTLV-1 proteins. These approaches have been shown to induce strong immune responses against HTLV-1 in animal models. It is important to note that while preclinical studies are promising, the safety and efficacy of these approaches in humans is not well known. Further clinical trials are needed to determine the safety and effectiveness of HTLV-1 vaccines in humans.

## Supplementary Information

**Additional file 1: Supplementary Table 1.** PRISMA 2020 checklist. **Supplementary Table 2.** Search strategies for online databases. **Supplementary Table 3.** Full characteristics of the included studies.

## Data Availability

Data sharing is available by contacting corresponding author.

## Abbreviations

HTLV-1: Human T-cell lymphotropic virus type 1
ATLL: Adult T-cell leukemia/lymphoma
HAM/TSP: HTLV-1-associated myelopathy/tropical spastic paraparesis
GP: Glycoprotein peptide
aa: Amino acid
nor-MDP: N-acetyl glucosamine-3yl-acetyl-L-alanyl-D-isoglutamine
CTL: Cytotoxic T Lymphocytes
ODN: Oligodeoxynucleotides
TNF: Tumor necrosis factor
IFN-γ: Interferon gamma
HLA: Human leukocyte antigen
CMV: Cytomegalovirus
Ig: Immunoglobulin
