# Supplementary material for: Human T-cell lymphotropic virus type 1 (HTLV-1) proposed vaccines: a systematic review of preclinical and clinical studies
Source: BMC Infect Dis. 2023 May 11;23:320. doi: 10.1186/s12879-023-08289-7 (PMC10173209; doi:10.1186/s12879-023-08289-7)
Supplement: Supplementary file 1 — Additional file 1: Supplementary Table 1. PRISMA 2020 checklist. Supplementary Table 2. Search strategies for online databases. Supplementary Table 3. Full characteristics of the included studies. [file 12879_2023_8289_MOESM1_ESM.docx]

# Supplementary Material

**Human T-cell lymphotropic virus type 1 (HTLV-1) proposed vaccines: A systematic review of preclinical and clinical studies.**

**Supplementary Table 1.** PRISMA 2020 checklist

**Supplementary Table 2.** Search strategies for online databases

**Supplementary Table 3.** Full characteristics of the included studies.

This supplemental material has been provided by the authors to give readers additional information about their work.

**Supplementary Table 1.** PRISMA 2020 checklist

| **Section and Topic** | **Item #** | **Checklist item** | **Location where item is reported** |
| --- | --- | --- | --- |
| **TITLE** | | |  |
| Title | 1 | Identify the report as a systematic review. | Page 1 |
| **ABSTRACT** | | |  |
| Abstract | 2 | See the PRISMA 2020 for Abstracts checklist. | Page 2 |
| **INTRODUCTION** | | |  |
| Rationale | 3 | Describe the rationale for the review in the context of existing knowledge. | Page 4 |
| Objectives | 4 | Provide an explicit statement of the objective(s) or question(s) the review addresses. | Page 4 |
| **METHODS** | | |  |
| Eligibility criteria | 5 | Specify the inclusion and exclusion criteria for the review and how studies were grouped for the syntheses. | Page 5 |
| Information sources | 6 | Specify all databases, registers, websites, organisations, reference lists and other sources searched or consulted to identify studies. Specify the date when each source was last searched or consulted. | Page 5 |
| Search strategy | 7 | Present the full search strategies for all databases, registers and websites, including any filters and limits used. | Page 5 |
| Selection process | 8 | Specify the methods used to decide whether a study met the inclusion criteria of the review, including how many reviewers screened each record and each report retrieved, whether they worked independently, and if applicable, details of automation tools used in the process. | Page 5 |
| Data collection process | 9 | Specify the methods used to collect data from reports, including how many reviewers collected data from each report, whether they worked independently, any processes for obtaining or confirming data from study investigators, and if applicable, details of automation tools used in the process. | Page 5 |
| Data items | 10a | List and define all outcomes for which data were sought. Specify whether all results that were compatible with each outcome domain in each study were sought (e.g. for all measures, time points, analyses), and if not, the methods used to decide which results to collect. | Page 5+ Table 1 to 5 |
|  | 10b | List and define all other variables for which data were sought (e.g. participant and intervention characteristics, funding sources). Describe any assumptions made about any missing or unclear information. | Page 5+ Table 1 to 5 |
| Study risk of bias assessment | 11 | Specify the methods used to assess risk of bias in the included studies, including details of the tool(s) used, how many reviewers assessed each study and whether they worked independently, and if applicable, details of automation tools used in the process. | N/A |
| Effect measures | 12 | Specify for each outcome the effect measure(s) (e.g. risk ratio, mean difference) used in the synthesis or presentation of results. | N/A |
| Synthesis methods | 13a | Describe the processes used to decide which studies were eligible for each synthesis (e.g. tabulating the study intervention characteristics and comparing against the planned groups for each synthesis (item #5)). | N/A |
|  | 13b | Describe any methods required to prepare the data for presentation or synthesis, such as handling of missing summary statistics, or data conversions. | N/A |
|  | 13c | Describe any methods used to tabulate or visually display results of individual studies and syntheses. | N/A |
|  | 13d | Describe any methods used to synthesize results and provide a rationale for the choice(s). If meta-analysis was performed, describe the model(s), method(s) to identify the presence and extent of statistical heterogeneity, and software package(s) used. | N/A |
|  | 13e | Describe any methods used to explore possible causes of heterogeneity among study results (e.g. subgroup analysis, meta-regression). | N/A |
|  | 13f | Describe any sensitivity analyses conducted to assess robustness of the synthesized results. | N/A |
| Reporting bias assessment | 14 | Describe any methods used to assess risk of bias due to missing results in a synthesis (arising from reporting biases). | N/A |
| Certainty assessment | 15 | Describe any methods used to assess certainty (or confidence) in the body of evidence for an outcome. | N/A |
| **RESULTS** | | |  |
| Study selection | 16a | Describe the results of the search and selection process, from the number of records identified in the search to the number of studies included in the review, ideally using a flow diagram. | Page 6 |
|  | 16b | Cite studies that might appear to meet the inclusion criteria, but which were excluded, and explain why they were excluded. | Figure 1 |
| Study characteristics | 17 | Cite each included study and present its characteristics. | Page 6 |
| Risk of bias in studies | 18 | Present assessments of risk of bias for each included study. | N/A |
| Results of individual studies | 19 | For all outcomes, present, for each study: (a) summary statistics for each group (where appropriate) and (b) an effect estimate and its precision (e.g. confidence/credible interval), ideally using structured tables or plots. | Table 1 to 5 |
| Results of syntheses | 20a | For each synthesis, briefly summarise the characteristics and risk of bias among contributing studies. | N/A |
|  | 20b | Present results of all statistical syntheses conducted. If meta-analysis was done, present for each the summary estimate and its precision (e.g. confidence/credible interval) and measures of statistical heterogeneity. If comparing groups, describe the direction of the effect. | N/A |
|  | 20c | Present results of all investigations of possible causes of heterogeneity among study results. | N/A |
|  | 20d | Present results of all sensitivity analyses conducted to assess the robustness of the synthesized results. | N/A |
| Reporting biases | 21 | Present assessments of risk of bias due to missing results (arising from reporting biases) for each synthesis assessed. | N/A |
| Certainty of evidence | 22 | Present assessments of certainty (or confidence) in the body of evidence for each outcome assessed. | N/A |
| **DISCUSSION** | | |  |
| Discussion | 23a | Provide a general interpretation of the results in the context of other evidence. | Page 16 |
|  | 23b | Discuss any limitations of the evidence included in the review. | Page 17 |
|  | 23c | Discuss any limitations of the review processes used. | Page 17 |
|  | 23d | Discuss implications of the results for practice, policy, and future research. | Page 17-18 |
| **OTHER INFORMATION** | | |  |
| Registration and protocol | 24a | Provide registration information for the review, including register name and registration number, or state that the review was not registered. | N/A |
|  | 24b | Indicate where the review protocol can be accessed, or state that a protocol was not prepared. | N/A |
|  | 24c | Describe and explain any amendments to information provided at registration or in the protocol. | N/A |
| Support | 25 | Describe sources of financial or non-financial support for the review, and the role of the funders or sponsors in the review. | None |
| Competing interests | 26 | Declare any competing interests of review authors. | None |
| Availability of data, code and other materials | 27 | Report which of the following are publicly available and where they can be found: template data collection forms; data extracted from included studies; data used for all analyses; analytic code; any other materials used in the review. | By contacting the corresponding author |

**Supplementary Table 2.** Search strategies for online databases

**Pubmed:453, Scopus:631, WOS: 607, total=1690, dup remove= 1250,**

**Cochrane:10**

| Database | Search strategy |
| --- | --- |
| Medline (PUBMED) | (((("Human T-lymphotropic virus 1"[Mesh]) OR (Human T-lymphotropic virus 1[Title/Abstract])) OR (Human T lymphotropic virus 1[Title/Abstract])) OR (HTLV*[Title/Abstract])) AND ((((("Vaccines"[Mesh]) OR (vaccines[Title/Abstract])) OR (vaccin*[Title/Abstract])))) |
| Web of Science | **(TOPIC:**(Human T-lymphotropic virus 1) *OR* **TOPIC:**(HTLV*) *OR TOPIC***:**(Human T lymphotropic virus 1) *Indexes=SCI-EXPANDED, SSCI Timespan=All years*  AND **TOPIC:**(vaccines) *OR* **TOPIC:**(vaccin*) *Indexes=SCI-EXPANDED, SSCI Timespan=All years )* |
| Scopus | ( ( TITLE-ABS-KEY ( vaccines ) OR TITLE-ABS-KEY ( vaccin* ) ) ) AND ( ( TITLE-ABS-KEY ( human AND t-lymphotropic AND virus 1 ) OR TITLE-ABS-KEY ( htlv* ) OR TITLE-ABS-KEY ( human AND t AND lymphotropic AND virus 1 ) ) ) |
| Cochrane library | ((Human T-lymphotropic virus 1):ti,ab,kw OR (HTLV*):ti,ab,kw (Word variations have been searched) AND (vaccines):ti,ab,kw OR (vaccin*):ti,ab,kw) |

**Supplementary Table 3.** Full characteristics of the included studies.

| **Author** | **Year** | **Country** | **Type of study** | **Number of participants** | **Host** | **Vaccine type** | **vaccine construct** | **Vaccine dose** | **Vector** | **Route** | **Prescribed number** | **Adjuvant** | **Laboratory method** | **Main findings** |
| --- | --- | --- | --- | --- | --- | --- | --- | --- | --- | --- | --- | --- | --- | --- |
| Ando, S. | 2017 | Japan | in vivo | NA | Three- to six-week-old female rats (F344/N Jcl-rnu/+) | Epitope–Pulsed Dendritic Cell | HTLV-1 Tax(180-188)-specific CTL epitope-pulsed dendritic cell therapy | 10^6 cells | NA | SC | once a week for 3 wk into rats | NA | PCR, ELISA, Flow cytometry, | monocyte-derived DCs capacity to stimulate CMV-specific autologous CTLs in vitro, peptide-pulsed DC immunotherapy will be useful to induce functional HTLV-1–specific CTLs and decrease PVL in infected individuals with high PVL and impaired HTLV-1–specific CTL responses, therby reducing the risk of the development of ATL. |
| Arp, J. | 1993 | USA | in vivo | NA | BALB/c (Charles River), C57BL/6 (CharlesRiver) and CFW/D (Ball & McCarter, 1979) | recombinant baculovirus vector | envelope protein inclusion bodies (env-I.B.) in the presence or absence of an adjuvant | 10 µg | baculovirus | IP | 10 µg of env-I.B at 6 and 8 weeks of age then boosed 2 and 4 weeks later | with/without an adjuvant formulated from a mycobacterial cell wall extract (MCWE; Bioniche/Vetrepharm) | Western blotting, radioimmunoprecipitation, peptide ELISA and a syncytium inhibition assay | Antibodies against the HTLV-I env protein in the presence or absence of an adjuvant, neutralizing Ab in admission of high doses of mycobacterial cell wall extract, enhanced Ab response to the HTLV-I envelope glycoprotein following priming with recombinant vaccinia virus (Rvv) constructs expressing either the entire native HTLV-I envelope (gp46 and gp21) or just gp46, Increased titres of neutralizing Ab following priming with the Rvv expressing gp46 only |
| Baba, E. | 1995 | Japan | in vivo | 12 (rabbits: 9 complete vaccine/3 just adjuvant) 15 (rat: 10 complete vaccine/5 just adjuvant) | Female New Zealand White rabbits and inbred female WWQdj, Fisher 433 (F433)/Qdj rats, Inbred female BN/Sea,  LewisiSea rats, d ACI/Jcl rats | peptide-based vaccines | 2 vaccines: T and B cell epitope-based peptide vaccine constructed from the conjugation of gp46 (aa 181-210) and (181-203) with a branched polylysine oligomer | 500 µg (rabbit) 100 µg (rat) | NA | IM/SC | 9 New Zealand White rabbits were immunized 500 µg of pKA- or OVA-conjugated peptide (MAP181-203, MAP181- 210, 181-2030VA) emulsified with CFA on day 0 and then with IFA on days 14 and 28. 5 different inbred strains of rats similarly immunized either i.m. or S.C. with 100 pg of pKA-conjugated peptide (MAP181-203,MAP181-210) emulsified with CFA on day 0 and then with IFA on days 14 and 28. Control rabbits and rats were immunized with the same amounts of CFA and IFA only. | peptides with a branched polylysine oligomer (pKA) | western blotting, ELISA, PCR, IF | neutralizing Ab production in rabbits ( X4 -8 and X8-64 titers in response to MAPl 81-203 and MAPl 81 -21 0, respectively), neutralizing Abs (X40 to X320) in five different strains of rats in response to MAP1 81-210 |
| Frangione-Beebe, M. | 2001 | USA | in vivo | 6 | female New Zealand white rabbits (12 weeks) | encapsulated or free peptide vaccine | vaccine (MVFMF2) comprising HTLV-1-gp46 (aa 175–218) linked by GPSL turn to MVF (aa 288–302) | Two rabbits: 18 mg of microspheres containing 1mg of peptide, and 3.7 mg of microspheres containing 100 microg of adjuvant (nor-MDP) Two additional rabbits: 18 mg microspheres containing 1 mg of peptide and no adjuvant. Microspheres containing peptide or adjuvant were suspended in 1 ml of 4:1 squalene-arlacel A (Sigma) and injected intramuscularly  remaining two rabbits: 1 mg of free peptide (1.3 mg/mL in PBS) and 100 mg of nor-MDP emulsied 50:50 in 4:1 squalene: arlacel A + boosting with 500 mg of peptide and 100 mg of nor-MDP at 10 weeks. | NA | IM | 4 rabbits once in 12 weeks  2 rabbits in 10 and 12 weeks | with or without N-acetyl-glucosamine- 3yl-acetyl-l-alanyl-d-isoglutamine (nor-MDP) adjuvant | ELISA, HPLC, Gun scanning electron microscope, | sustained antibody response over a period of 5 months, without requiring a booster immunization or adjuvant in response to encapsulation of MVFMF2, elevated immune response invoked by the encapsulated peptide without requiring booster and adjuvant, Raised Ab level against both free and encapsulated MVFMF2 |
| Grange, M. P. | 1997 | France | in vivo | 18 | 6 to 8 week old male BALB/c mice | genetic and protein inoculation (gp62 Baculovirus recombinant protein + vector) | complete HTLV-I envelope | 10 microg for protein | 7 mice: CMVenvLTR plasmid 7 mice: CMVenv plasmid 4 mice: negative control plasmid (pcDNA3) | IM (vector)/IP(protein) | [*different protocls were used] Mice were immunized IP with 10 µg of gp62 Baculovirus recombinant protein in complete Freund's adjuvant followed by three boosting doses of 10 microg of recombinant protein in incomplete Freund's adjuvant at 2-week intervals. Two mice of each DNA-primed group were immunized with protein at 14 weeks post-DNA inoculation. For comparative studies, two naive mice were immunized with protein with the same protocol | Freund's adjuvant | ELISA, IFA, syncytium formation assay, CTLL assay | high antibody response in response to protein boosts in mice primed with DNA expressing HTLV-I envelop proteins, high neutralizing antibody titers, memory B-cell clone stimulation via single inoculation of DNA expressing HTLV-I env gene, specific cellular helper cell response in mice. |
| Hanabuchi, S. | 2001 | Japan | in vivo | 2 (vaccinated) 2 (control) | Four-week-old female F344/N Jcl-rnu/rnu (nu/nu or athymic) rats and F344/N Jcl-rnu/+ (nu/+) rats | peptide-based vaccine | synthetic oligopeptides corresponding to the Tax-epitope(180-188) | 100 microg Tax 180–188 peptide alone, 10 nmol of ISS-ODN alone, 100 microg Tax 180–188 peptide mixed with 10 nmol of ISS ODN (Tax 180–188/ISS-ODN), or 100 microg Influenza A matrix 58–66 peptide mixed with 10 nmol of ISS-ODN (Influenza A matrix 58–66/ISS-ODN) | NA | ID/IP | twice with a 2-week interval Two weeks after the last immunization, 107 freshly isolated T-cell enriched splenocytes from vaccinated rats were intraperitoneally inoculated into 4-week old nu/nu rats, which were simultaneously inoculated subcutaneously with 2×10*7 FPM1-V1AX cells | immune–stimulatory DNA sequences–oligodeoxynucleotides (ISS-ODNs) | cold inhibition assay/Cr-release assay/peptide mapping | development prevention of FPM1-V1AX cell induced lymphomas in athymic rats in response to adoptive transfer of the Tax 180–188-specific CTL line or freshly prepared T cells from rats vaccinated with the Tax 180–188 oligopeptide in comparison with control groups,  equivalent inhibitory effects on the growth of HTLV-I-infected tumors in both CD4+ and CD8+ T cells, Tax 180–188 as a dominant epitope recognized by the HTLV-I Tax-specific CTL line |
| Ibuki, K. | 1997 | Japan | in vivo | 5 | cynomolgus monkeys (Macaca fascicularis) | rVV | HTLV-I envelope (Env) gp46 | Two monkeys: 3.10*8 p.f.u. of WR-SFB5env three monkeys: 3.10*8 p.f.u. of HA− VV | plasmid pSFB5, which contains the A-type inclusion body (ATI) promoter of cowpox virus and five units of synthetic VV 7±5 kDa early promoter | ID | 1 | Freund’s complete adjuvant | western blot, PCR, particle agglutination, IFA | Neither HTLV-I antigen nor HTLV-I proviruses were detected/ Single immunization with WR-SFB5env elicited long-lived anti-Env antibodies as well as Env-specific CTL activity/ Gp46 expression alone was sufficient for protection |
| Kuo, C. W. | 2011 | Scotland | NA | NA | NA | recombinant glycoprotein | gp46 | soluble recombinant surface glycoprotein (gp46, SU) fused to the Fc region of human IgG (sRgp46-Fc) | pHTE-1, pMtgp46-Fc | NA | NA | NA | ELISA, Western blot, Syncytium interference assay, Flow cytometry, | High titer Ab responses/ Many of these mAbs recognize envelope displayed on the surface of HTLV-1–infected cells / mAbs robustly antagonize envelope-mediated membrane fusion and neutralize pseudovirus infectivity/ Potent neutralizing mAbs recognize the N-terminal receptor-binding domain / Both neutralizing and poorly neutralizing Abs strongly stimulate neutrophil-mediated cytotoxic responses to HTLV-1–infected cells. |
| Nakamura, Hideo | 1987 | Japan | in vivo | 10 | Cynomolgus monkeys (Macaca fascicularis) | hyprid protein | env gene products of HTLV-I produced in E.coli | different (100 or 150 microg) | NA | ID/IV | different for each group | Freund’s complete adjuvant | SDS-polyacrylamide gel electrophoresis, Western blot, IFA | Ab against HTLV-I gp68 and gp46, strong inhibition of syncytium formation, humoral immunity |
| Ohashi, T. | 2000 | Japan | NA | NA | Female F344/N Jcl-rnu/rnu (nu/nu) rats and F344/N Jcl-rnu/1 (nu/1) rats | DNA vaccine | Tax-coding DNA | 10 μg | Wild-type Tax (pbMT-2Tax) and a mutant Tax (Tax410) | The Helios Gene Gun system | twice, with a 1-week interval | Fifty milligrams of Au particles with 100 mg vector | Cr-release cytotoxicity assay, SDS-PAGE, | Tax-specific CTL induction, CTLs ability to lyse HTLV-1 infected syngeneic T cells in vitro, in vivo growth inhibition of HTLV-1-transformed tumor, efficient anti-tumor immunity induction |
| Sagar, Divya | 2014 | USA | in vivo | NA (exact number is not mentioned) | Transgenic hybrid mice generated from an intercross between HLA-A2.1 and DTR transgenic mice / HLA-A2.1 transgenic mice / DTR transgenic mice the last two types were used to produce the hybrid mice | dendritic cell-based anti-HTLV-1 vaccine | Tax(11-19) epitope | 100 μg | NA | ID/SC | once | absence or presence of Freund’s adjuvant and/or DCs | PCR/ELISA/MILLIPLEX magnetic bead assay | reduced proliferation of CD8+ splenocytes from Tax 11-19 immunized DC depleted mice, higher frequency of Tax 11- 19-specific cells with adjuvant usage, Tax 11-19 epitope as a potential candidate for a DC-based anti-HTLV-1 vaccine |
| Shida, H. | 1988 | Japan | in vivo | NA | Japan albino rabbits, each weighing 1.8 to 2.3 kg and 5-week-old male DDY mice | rVV | HTLV-1 envelope gene | NA | LC16mO not exactly described ( p7.5 promoter and flanked with the segments of the HA gene) | IP/IC | once | NA | IFA | LC16mO is a good candidate as a vector for vaccination |
| Shida, H. | 1987 | Japan | in vivo | 12 | rabbits | rVV | The envelope gene of HTLV-I in the vaccinia virus hemagglutinin (HA) gene | NA | WR-envl7 and WR-proenvl. | ID | once | NA | IFA | HA gene is a useful site to accept and express foreign genes/ A single inoculation of the recombinant virus-induced antibodies to the env proteins of HTLV-I in rabbits and had a protective effect against HTLV-I infection. |
| Suehiro, Youko | 2015 | Japan | human | 3 | human | autologous dendritic cells | autologous dendritic cells (DCs) pulsed with Tax peptides corresponding to the CTL epitopes | 5*(10^6) | NA | SC | three times at 2-week intervals | autologous dendritic cells (DCs) induced from the peripheral monocytes. | PCR | Tax specific CTL response, partial remission in 1 patient, complete remission in 1 patient, maintaining remission status without any additional chemotherapy, progressive disease in 1 patient, |
| Sugata, Kenji | 2015 | Japan | in vivo | 2 monkeys NA (mice) | Ly5.1 C57BL/6 mice, rhesus monkeys | rVV | HTLV-1 basic leucine zipper (bZIP) factor (HBZ) or Tax | Each animal received a dose of 107 plaque forming units of rVV in 10 mL of viral suspension. | pBMSF7c plasmid | skin sacrifation | In mice, 4 weeks after the first vaccination, 5 booster vaccinations were administered every 3 weeks In monkeys, booster vaccinations were repeated every 4 weeks. PBMCs from monkeys were obtained every 2 weeks. | cytosine phosphate guanine (with peptide) | ELISPOT, immunoblotting | Increased survival of the lymphoma cell–inoculated mice/ Induction of specific T-cell responses to HBZ and Tax in HTLV-1–infected rhesus monkeys/ A candidate peptide (HBZ157-176) for vaccine development was identified/ Dendritic cells pulsed with this peptide could generate HBZ-specific CTLs from human CD81 T cells. |
| Sundaram, R. | 2004 | USA | in vivo | NA  (exact number is not mentioned) | leukocyte antigen–A*0201 monochain trans genic H-2Db -2m double-knockout (HHD) mice | Multiepitope T-cell peptide vaccine | multivalent cytotoxic T-lymphocyte peptide construct derived from the Tax protein ofHTLV-1 separated by arginine spacers | 100 µg of multiepitope peptide or with a mixture of 33 µg of each of the 3 individual epitopes combined with 140 µg of TT3, a pro miscuous T-helper epitope from tetanus toxoid (residues 947–967) and 100 µg of adjuvant N-acetyl-glucosamine-3-acetyl L-alanyl-D-isoglutamine (nor-MDP; Peninsula Laboratories, Belmont, CA)22 emulsified 50:50 in 4:1 squalene/Arlacel A (Sigma, St. Louis, MO). | NA | SC | twice, 3 weeks apart | N-acetyl-glucosamine-3-acetyl L-alanyl-D-isoglutamine | reverse-phase high-performance liquid chromatography (RP-HPLC), Cr Release Assay, interferon-gamma Release Assay, ELISA, Plaque Assay for Viral Titers | significant reduction in viral replication dependent on CD8+T cells |
| Sundaram, R. | 2003 | USA | in vivo | NA | transgenic HHD mice | peptide vaccine | three HLA-A∗0201 restricted CTL epitopes (Tax11–19, Tax178–186, and Tax233–241) | 100 g of peptide mixed with 140 g TT3 | NA | SC | twice, 3 weeks apart | 100 microg N-acetyl-glucosamine-3-acetyl-l-alanyl d-isoglutamine (nor-MDP) | ELISPOT, Cr release assay | cellular responses to each intended epitope in vivo, high level of IFN-γ production |
| Ishizawa, M. | 2021 | Japan | in vitro | NA | NA | Mitomycin C-treated HLA-A2-negative HTLV-1-infected T-cell lines or short-term cultured peripheral blood mononuclear cells (PBMC)) | Tax | NA | NA | NA | NA | NA | ELISA, PCR, CTL assay | Short-term cultured autologous PBMC from ATL patients could potentially serve as a vaccine to evoke Tax-specific CTL responses. |
| Amirnasr, M. | 2016 | Iran | in vivo | 70 | male BALB/c mice | recombinant protein | env23 (162-209 ) and env13 (125-209) recombinant proteins | 7.5 µg antigen | pET102/D vector (and transferred into Escherichia coli strain TOPO10) | nasal/SC | 3 immunizations (7.5 µg antigen) were performed with 2 weeks intervals | CHT and TMC nanoparticles | ELISA, PCR | higher serum IgG1 and IgG total levels compared to antigen solution, higher IgG2a levels and IgG2a/IgG1 ratio in nasal delivery compared with subcutaneous administration (P < 0.001), higher cellular immune responses in response to env23 antigen, compared with env13. |
| Armand, M. A. | 2000 | France | in vivo | 40 (10 were control) | female BALB/c mice | DNA vaccine | two types of plasmids for DNA: 1) coding DNA of the complete env gene of HTLV-I under the control of the CMV promoter with (CMVenvLTR) or without (CMVenv) the tax/rex genes, 2) coding DNA of the complete env gene of HTLV-I under the control of the human desmin muscle specific promoter (DesEnv) | 100 microg | CMVenvLTR/CMVenv/DesEnv/pcDNA3/DesEnvDNϪco the last two were used for control groups (group 4 and 5) | IM | 3 immunizations were performed with 2 weeks intervals | NA | PCR, Flow-cytometry, ELISA and neutralization assays | detectable and neutralizing humoral response, higher humoral response with better neutralization properties in response to the DesEnv construct compared to CMVenvLTR or CMVenv plasmids |
| Arp, J. | 1996 | USA | in vitro | NA | baculovirus non-fusion vector system | rVV | gp46 | NA | pTME-46 | NA | NA | mycobacterial cell wall extract | PCR, ELISA, western blot, y immunofluorescence assays | Maintenance of highly conserved conformational epitopes in the recombinant HTLV-1 envelope protein structure |
| Ford, C. M. | 1992 | USA | in vivo | 15 | Balb/c, A/J, and C57BU6 strains of mice | Recombinant vaccinia viruses | RVV El expressed the native HTLV-I envelope proteins gp46 (surface protein) and gp21 (transmembrane protein) RVV E2 expressed the envelope precursor with the proteolytic cleavage site deleted RVV E3 construct expressed only the external surface glycoprotein (gp46) | NA | pSC11 | intraperitoneal | NA | NA | Southern blot, Immunofluorescence assays, Radioimmunoprecipitation assays, ELISA, Western blot assays | Balb/c mice responded poorly to immunization with all of the three RVV constructs. C57BU6 mice produced neutralizing antibodies in response to immunization with all three constructs, whereas A/J mice developed neutralizing antibodies only when immunized with the RVV El s construct. The results indicate that the humoral immune responses depend on the form of HTLV-I envelope proteins expressed by each RVV. |
| Franchini, G. | 1995 | USA | in vivo | 12 | New Zealand White rabbits | live recombinant vaccine | gp63 | 10^7 plaque-forming units [PFU] | ALVAC | IM | 2 immunizations were performed with 1 month interval | Alum | IFA, PCR, syncytia inhibition assay | The results indicated that two inoculations of the ALVAC-based HTLV-1 env vaccine candidate protected animals against viral challenge 5 months following the last immunization |
| Frangione-Beebe, M. | 2000 | USA | in vivo | 12 rabbits 9 mice | Outbred female ICR mice  female New Zealand white rabbits | recombinant protein | MVFMF2 comprising HTLV-1-gp46 (aa 175–218) linked by GPSL turn to MVF (aa 288–302) | 1 mg of peptide | pT7-7 | IM | different for host groups | N-acetyl-glucosamine-3yl-acetyl-L-alanyl-D-isoglu tamine, nor-MDP | PCR, SIA, ELISA, western blot, circular dichroism (CD) spectroscopy | enhanced reactivity to viral antigens in rabbits, high titered anti-peptide antibodies in mice, immunogenic in an outbred population of both rabbits and mice when administered with adjuvant, enhanced immunogenicity when encapsulated in biodegradable microspheres without requiring of adjuvant, syncytium formation inhibition ability of anti-rabbit and anti-mouse Abs, no protection from cell-associated viral challenge in rabbits |
| Fujii, H. | 2016 | Japan | in vivo | 2 (both were pregnant) | Strains of SD rats | monoclonal antibody of rat origin (LAT-27) | gp46 amino acids 191–196 | 25 mg/head of either LAT-27 or isotype control mAb two times | NA | IP | two times on –7 d and –2 d of delivery | NA | ELISA, qPCR, Flow Cytometry, SIA | When humanized immunodeficient mice were pre-infused intravenously with humanized LAT-27 (hu-LAT-27), all the mice completely resisted HTLV-I infection. These results indicate that hu-LAT-27 may have a potential for passive immunization against both horizontal and mother-to-child vertical infection with HTLV-I. |
| Hakoda, E. | 1995 | Japan | in vivo | 6 | Japanese white rabbits | recombinant vaccinia virus | env gene in the hemagglutinin locus, WR-SFBSenv | 1 x 10^8 plaque-forming units of recombinant or control virus at 3 sites on the back. | NOT EXACTLY DESCRIBED (recombinant vaccinia virus containing the entire HTLV-I env gene in the hemagglutinin locus, WR-SFBSenv, in which the env gene was expressed under the control of the hybrid promoter (Funahashi et al., 1991), will be described in detail. Vaccinia virus lacking the functional hemagglutinin gene, HA WR, was used as control (Shida etal., 1987). ) | intradermally | one time in 3 sites in the back of tabbits | NA | PCR, western bloth, ELISA, plaque-reduction assay | Incapable of inducing neutralizing antibodies |
| Kabiri, M. | 2018 | Iran | in vivo | NA | BALB/c male mice | chimeric peptide vaccine | chimeric peptide vaccine including Tax, gp21, gp46, and gag immunodominant epitopes of human T-cell lymphotropic virus type 1 (HTLV-1) | 10 microg | pET32b (+) plasmid the synthetic nucleotide sequence of chimera was cloned into pET32b (+) plasmid vector and expressed in E. coli BL21(DE3) host | nasal/SC | three times at two weeks intervals | monophosphoryl lipid A (MPLA) or ISCOMATRIX (IMX) | ELISA, PCR | increased Ab titers containing IgG2a, mucosal IgA, as well as IFN-γ and IL-10 cytokines and decreased TGF-β1 level in response to mixture of IMX and chimera, potent mucosal sIgA titers in intranasal delivery compared to subcutaneous root, cell-mediated responses, as evident by higher IgG2a and IFN-γ, as well as suppressed TGF-β1 level in SC or nasal delivery |
| Kabiri, M. | 2018 | Iran | in vivo | NA | BALB/c male mice | fusion epitope-loaded PLGA nanoparticles (NPs) | chimeric peptide vaccine including Tax (aa 11-19 and aa 178-186), gp21 (aa 370-400), gp46 (aa 165-306), and p19 (aa 105-124) immunodominant epitopes of human T-cell lymphotropic virus type 1 (HTLV-1) | 10 microg | pET32b (+) plasmid vector (Novagen, USA) | nasal/SC | three times at two weeks intervals | with or without CpG adjuvant | ELISA, PCR, western blot | elevated titers of IgG1, IgG2a, and sIgA antibodies, as well as IL-10, and IFN-γ cytokines and decreased TGF-β1 level, promoted cellular and mucosal responses in co-delivery of chimera and CpG ODN in PLGA |
| Kazanji, M. | 1997 | France | in vivo and in vitro | 25 (not mentioned exactly, the number is extracted from table II) | WKY and Fischer F-344 rats | recombinant adenovirus-5 DNA plasmids and vaccina virus | The complete human T-cell leukemia virus type I (HTLV-I) env gene was inserted into an expression cassette containing the adenovirus 5 major late promoter (Ad5-MLP). (Recombinant Ad5-HTLV-I-env) | Fischer F-344 rats: 107 PFU of WR-SFB5 env or control HA-WR/ WKY rats: 200 µl PBS containing 109 PFU of Ad5-HTLV-I-env (or Ad5-HTLV-I-gp46 for boosting) or 100 µg of the naked DNA expression vector pMLP-HTLV-I-env. Booster injections with baculovirus-derived recombinant gp46 (1 µg) were delivered subcutaneously together with 50 µg of saponin as adjuvant | recombinant vaccinia vector WR-SFB5env, adenovirus vector | IM/intradermally | different for host groups | saponin (QuilA, Superfos, Denmark) as adjuvant | IFA, western blot, PCR, SIA, CTL assay | WKY rats: No detectable Ab against HTLV-I, recovery of HTLV-I-specific cytotoxic T lymphocytes in all immunized groups but not from controls, Fischer F-344 rats: Ab against the HTLV-I env gp21 and gp46 (non-neutralizing), partial protection in both immunization regimens after challenge with human HTLV-I-producing cells (MT-2) |
| Kazanji, M. | 2006 | France | in vivo | 5 | male squirrel monkeys | Chimeric peptide vaccine | three HLA-A*0201-restricted CTL epitopes derived from Tax protein (Tri-Tax) and B-cell env epitope (aa 175–218) | 700 microg | NYVAC | IM | Two monkeys were injected twice, at 0 and 4 weeks, with the Env B-cell epitope aa 175–218 (500 mg per monkey) linked to the promiscuous T-helper cell epitope MVF (700 mg per monkey), as described pre viously (Frangione-Beebe et al., 2000). Six weeks after the first immunization, the monkeys were injected with another construct, consisting of the three Tax CTL epitopes (aa 11–19, 178–186, 306–315). Monkeys were boosted twice at weeks 9and 16 with both B- and T-cell epitopes. | N-acetylglucosamine-3yl-acetyl-L-alanyl-D-isoglutamin | ELISA, PCR, western blot | high titre of Abs, high frequency of specific IFN-c-producing cells and partial protection |
| Kazanji, M. | 2001 | France | in vivo | 8 | male squirrel monkeys | vaccinia virus-derived NYVAC | env/gag | In the initial protocol, three monkeys 10^8 PFU of NYVAC / Six months after the last administration of NYVAC-env, two of the three vaccinated monkeys were boosted with 500 mg of the naked DNA immunogen CMV-env-LTR, The third monkey and the control were injected with a naked DNA vector containing the b-galactosidase gene (CMV-bgal) In the second immunization protocol, three monkeys 500 mg of the DNA immunogen CMV-env-LTR and the control monkey was injected with the CMV-bgal vector. Six months later,the three vaccinated monkeys received a series of three booster injections, separated by 1-month intervals, of 108 PFU of the NYVAC-based candidate vaccine containing the HTLV-1 env and gag genes. The control monkey received 108 PFU of NYVAC-RG at the same times | CMV-ßga | IM | protocol A: (3 monkeys) 0, 1, and 3 months (108 PFU of NYVAC containing the HTLV-1 env gene) Six months after the last administration of NYVAC-env, two of the three vaccinated monkeys were boosted with 500 mg of the naked DNA immunogen CMV-env-LTR intramuscularly into the tibialis anterior muscle protocol B: (3 monkeys) 500 mg of the DNA immunogen CMV-env-LTR. Six months later, received a series of three booster injections, separated by 1-month intervals, of 108 PFU of the NYVAC-based candidate vaccine contain ing the HTLV-1 env and gag genes. | NA | ELISA, PCR, western blot | protocol A: With the first immunization protocol, no anti-bodies against HTLV-1 HTLV-1 Env gp46 was stimulated in all of the three immu nized monkeys and a lesser response was stimulated in the control monkey protocol B: did not induce detectable levels of antibodies against HTLV-1 In the lymphocyte proliferation test performed 1 month after boosting, high-level, specific responses were detected in the three immunized animals against both recombinant Env gp46 protein and Gag peptides but not in the control monkey |
| Kozako, T. | 2009 | Japan | in vivo | NA | HLA-A*0201-transgenic mice | chimeric particle | HTLV-1/hepatitis B virus core (HBc) chimeric particle incorporating the HLA-A*0201-restricted HTLV1 Tax-epitope | HTLV-1/HBc chimeric particle (20microg), or Tax11–19 peptide / HBc particle (20microg) and peptide (1microg) | pUC18 (The chimeric genes were PCR amplified by using pUC18 DNA vector, the purified PCR product was digested with theappropriate enzymes, and ligated to the BamHI and EcoRI treated P. pastris expression vector, pPIC3.5 (Invitrogen BV, Gronin gen, The Netherlands). The chimeric protein was expressed in P. pastris KM71. | intradermally | days 0 and 14 with HTLV-1/HBc chimeric particle (20 microg), or Tax11–19 peptide | NA | ELISPOT, PCR, western blot, FCA, ELISA, enzyme-linked immunospot assay | induction of HTLV-1 Tax-specific CD8+ cells from spleen and inguinal lymph nodes after immunization, efficient induction of IFN- -producing cells, antigen-specific gamma-interferon reaction induction, increased expression of CD86, HLA-A02, TLR4 and MHC class II in dendritic cells, HTLV-1-specific CD8+ T-cells induction by peptide with HTLV-1/HBc particle from ATL patient, but not by peptide only, lysing cell presenting the peptide by HTLV-1-specific CD8+ T-cells |
| Kozako, T. | 2011 | Japan | in vivo | 5 | HLA-A*0201-transgenic mice | Oligomannose-coated liposomes | an HTLV-1-specific CD8+ T-cell response by oligomannose-coated liposomes (OMLs) encapsulating the human leukocyte antigen (HLA)A*0201-restricted HTLV-1 Tax-epitope (OML⁄Tax) | 1 microg | NA | intradermally | days 0 and 14 with OML⁄Tax, Tax peptide alone or phosphate-buffered saline (PBS). | OML | ELISPOT, FCM assay | resulted in the efficient induction of IFN-gamma-producing cells, induction of HTLV-1 Tax-specific CD8+ cells from inguinal lymph nodesafter immunization with OML/Tax, increased CD86,MHCI, HLA-A02 and MHCII levels upon exposure of dendritic cells to OML⁄Tax |
| sundaram | 2004 | USA | in vivo | 2 rabbits | Female New Zealand outbred white rabbits, female ICR mice (8 weeks) | peptid vaccine | chimeric synthetic B-cell epitopes derived from HTLV-1-Env (gp21 and gp46) with promiscuous T-helper epitopes derived either from the tetanus toxoid (amino acids 947–967) or measles virus fusion protein (amino acids 288–302) | rabbits: initial dose (1 mg of the chimeric peptide) + booster (500 µg of peptide) / mice: initial dose (100 µg of peptide) + booster (500 µg of peptide) | NA | s.c. in the thigh muscle in 2 rabbits, s.c in mice | rabbits [initial dose + booster injections every 3–5 weeks apart], mice [initial dose+ booster at 3 and 6 weeks] | nor-MDP adjuvant | Circular dichroism spectroscopy, computer-aided analyses of protein antigenicity, Syncytia inhibition assay, Flow cytometry, Immunogenicity testing, | Neutralizing Ab against the epitopes derived from the gp21, inhibition the formation of virus-induced syncytia, peptid had secondary structure correlated well with the crystal structure data or predicted structure |
| Laimore | 1995 | USA | in vivo and in vitro | 20 mice ( 5 per strain), 20 rabbits | Female inbred strains of mice (BALB/c, C3H/HeJ, and C57BL/6) and outbred ICR mice,and New Zealand White rabbits (12 weeks of age) | peptid vaccine | chimeric B- and T-cell epitopes of HTLV-1 env-gp46 (SP2 [aa 86 to 107] and SP4a [aa 190 to 209) with promiscuous T-cell epitopes (from tetanus toxin and MVF protein) | primary and booster dose after 3 weeks( 100 micro gr) in mice - primary and booster dose after 2 weeks( 500 micro gr) in rabbits | vector control protein SJ26 | subcutaneously | priming dose and booster after 3 weeks in mice/ 2 weeks in rabbits | not define the adjuvant's name | direct and competitive ELISA, radioimmunoprecipitation assay (RIPA), human osteosarcoma cell-based assay(SIA), syncytium inhibition assay, and antigen-induced lymphocyte proliferation assays | Ab production, inhibiton of HTLV-1-mediated syncytium formation, induction of lymphocyte proliferation in response to SP4a in mice, promotion of virus-specific helper T-cell responses |
| shafifar | 2022 | Iran | in vivo and in vitro | 15 | male 6 to 8 weeks pathogen-free BALB/c mice | recombinant protein | Fc-fusion multi-immunodominant recombinant protein (Tax-Env: mFcγ2a and Tax-Env: His ) | 1) Six mice received 50 μg of tTax-tEnv:mFcγ2a in 100 μL PBS + 100 μL of DDA adjuvant /2) Four mice, 50 μg of tTax-tEnv:His in 100 μL of PBS +100 μL of DDA adjuvant / 3) Five mice, 200 μL of PBS (negative control) | pPICZaA vector | intraperitoneal | T200 μL/mouse thrice at two-week intervals (0, 14th, and 28th days) | Dimethyl dioctadecyl ammonium bromide (DDA) | SDS-PAGE , Western blot, real time PCR | significant increase in IFN-γ and IL-12 release in response to Tax-Env: mFcγ2a compared to Tax-Env: His, 50% low proviral load of HTLV-1 and 50% complete protection in challenged mice, more Th1 immune responses in response to "Tax-Env: mFcγ2a”, more Th2 immune responses in response to “Tax-Env: His” |
| Jahantigh | 2021 | Iran | in silico | NA | NA | epitope-based vaccine | eight-epitopes-rich domain, including overlapping epitopes detected on both B and T cells constructed of Gag, Env, Pol, Hbz, and Tax proteins | NA | pET28a (þ) vector | NA | NA | NA | antigen prediction, Mapping, 3D Structure modeling, Homology modeling, Antigenicity and allergenicity and solubility and other physicochemical parameters evaluation, structure prediction, In silico cloning, Immune simulation, peptide–allele docking | interaction of the epitope and the designed protein with immune receptors(in silico docking), strong interaction of O2 epitope and D8 protein with immune receptors especially the HLAA 02:01 receptor, stability of the interactions for 100 ns(molecular dynamic ), root mean square deviation, radius of gyration, hydrogen bonds, and solvent-accessible surface area were calculated for the 100 ns, humoral and cell-mediated immune responses elicited |
| Alam | 2020 | Bangladesh | in silico | NA | NA | Epitope Ensemble Vaccine | prediction of 14 epitopes for targeting Glycoprotein 62 | NA | NA | NA | NA | NA | Variability Analysis of GP62 of HTLV-1, Population Protection Coverage (PPC) Calculation, HLA-Epitope Binding Prediction, Molecular Dynamics Simulation, Prediction ofB-Cell Epitope, | ALQTGITLV and VPSSSTPL epitopes interaction with three MHC alleles ( including HLA-A*02:03, and HLA-B*35:01, respectively ), 70% summative population protection coverage |
| Raza | 2021 | Bangladesh | in silico | NA | NA | Epitope-based universal vaccine | predicting HTLV-1 TAX multiepitope protein constructed from CTL and B cell epitopes | NA | pET-SUMO vector | NA | NA | PMISWPCPKD peptid adjuvant | Primary, secondary, tertiary and 3-D structure analysis, B- and T-cell epitope prediction, molecular docking analysis, Disulfide engineering, in silico cloning, | most antigenic score of 0.57, strong T cell epitopes interaction with HLA-A*0201, high binding affinity of the vaccine construct for TLR4 (in molecular docking study), most antigenic and immunogenic epitopes in in-silico investigation: B cell epitopes (KEADDNDHEPQISPGGLEPPSEKHFR and DGTPMISGPCPKDGQPS spanning from 324–349 and 252–268 respectively); T cell epitopes (LLFGYPVYV, ITWPLLPHV and GLLPFHSTL ranging from 11–19, 163–171 and 233–241) |
| Tariq | 2021 | Pakistan | in silico | NA | NA | multi-epitope-based subunit vaccine | 9 Cytotoxic T Lymphocytes, 6 Helper T Lymphocytes and 5 Linear B Lymphocytes epitopes, joint through linkers and adjuvant | NA | pET30a vector | NA | NA | β-defensin adjuvant | Conservation analysis and selection of predicted epitopes, Epitope modeling and molecular docking, homology analysis, Disulphide engineering , In-silico estimation and cloning, | strong binding affinity with their corresponding Human Leukocyte Antigen alleles, 95.8% coverage of the world’s population, highly antigenic properties while being non-toxic, soluble, non-allergenic, and stable in nature, enhanced stability via disulphide engineering, strong association between vaccine construct and human pathogenic immune receptor TLR3 (in Molecular docking analysis and Molecular Dynamics (MD)), rapid antigen clearance and higher levels of cell-mediated immunity in response to repeated-exposure and immune simulations, respectively. |
| Pandey | 2019 | India | in silico | NA | NA | multiepitope subunit vaccine | vaccine by the assimilation of B‐cell, CTL , and HTL epitopes for GAG, POL, ENV, P12, P13, P30, REX, and TAX proteins | NA | pET28a(+) expression vector | NA | NA | β-defensin adjuvant | B‐cell, Helper T‐cell (HTL), and Cytotoxic T‐cell (CTL) epitope prediction, Tertiary structure prediction, Molecular docking, in silico cloning | interactions with the HLA-A0201, HLA-A0701 and HLA-A0301 receptors, Strong interaction with TLR-3 |
| Mulherkar | 2018 | USA | in vivo and in vitro | NA | Six to eight-week old female HLA-A2 transgenic mice / Cell lines for in vitro investigations: HepG2, hepatoma cells, MT2, HTLV-1 virion expressing cells, and T2, TAP deficient lymphoblasts | peptid vaccine | MHC-I-bound HTLV-1 peptides | NA | NA | interadermal near the base of the tail and subcutaneous on the flank | Three injections: initial inoculation (consisted of a mixture of pooled free peptide in PBS plus Montanide ISA 51 (Seppic, Paris, France) (50:50 emulsion), PBS alone, or two independent, individual free peptide in PBS plus Montanide ISA 51 (50:50 emulsion)) + repeated two more times at 10-day intervals) | Montanide ISV-51 adjuvant | Degranulation assay, CD8+ T-cell killing assay, Mass spectrometry analysis, Flow cytometry analysis, ELISpot assays, MagPix cytokine detection, | confirmation of six novel MHC-I restricted epitopes capable of binding HLA-A2 and HLA-A24 alleles, generation of CD8+ T cells specific for each of these peptides, generation of epitope-specific CD8+ T cells secreted IFN-γ, granzyme B, MIP-1α, TNF-α, perforin and IL-10 in the presence of MT-2 cell line in vitro, cytotoxic response through surface expression of CD107 on CD8+ T cells when cultured with MT-2 cells, significant antiviral activity of CD8+ T cells specific against all identified peptides, In vivo generation of CD8+ T cells similarly demonstrated immunogenicity on ELISpot, CD107 degranulation assay, and MagPix MILLIPLEX analysis |
| Kobayashi | 2012 | Florida | in vitro | NA | Cell lines: EBV-LCLs, Mouse fibroblast cell lines (L cells), HTLV-1 infected T cell lymphoma cell lines, TL-Su, TCL-Kan,OKM-2T, Hut102, TL-Hir, Jurkat T cell lymphoma cell line, prostate tumor cell line PC3, and PBMCs | HLA-DR-bound peptide from the IL-9 receptor alpha of HTLV-1-transformed T cells | synthetic peptide corresponding to the identified IL-9Ra sequence | NA | NA | NA | NA | NA | Purification of HLA-DR molecules, Preparation of bound peptides, Binding assay, Western blot analyses, Cell-mediated cytotoxicity assays, | antigen-specific CD4 helper T lymphocytes generation (in vitro) restricted by HLA-DR15 or HLA-DR53 molecules with recognizing and killing ability of HTLV-1+, IL-9Ra+ T cell lymphoma cells |
| Kobayashi | 2006 | Florida | in vitro | NA | Cell lines: EBV-LCLs, Mouse fibroblast cell lines (L-cells), HTLV-I-infected T-cell lymphoma cell lines TL-Su, TCL-Kan, HUT102, TL-Hir (HTLV-1 Tax negative), and OKM-2T, Jurkat T-cell lymphoma cell line (HTLV-I negative), MT2, | peptid vaccine | Potential HLA-DR-restricted CD4+ T-cell epitopes of HTLV-1 Tax peptid | NA | NA | NA | NA | NA | Western blot analysis, ELISA , ECL detection system, chemiluminescence assay, cytokine release assay, high-performance liquid chromatography, mass spectrometry, | T-helper-cell induction in response to peptides Tax191–205 (restricted by the HLA-DR1 and DR9 alleles) and Tax305–319 (restricted by either DR15 or DQ9), Both these epitopes were naturally processed by HTLV-1+ T-cell lymphoma cells and by autologous APCs that were pulsed with HTLV-1Tax+ tumor lysates. These epitopes lie proximal to known CTL epitopes, which will facilitate the development of prophylactic peptide – based vaccine capable of inducing simultaneous CTL andT-helper responses |
| Schönbach | 1996 | Japan | in vitro and in vivo | NA (expressed: at least two mice were immunized with each peptide) | Eight- to ten-week-old transgenic HLA-B*3501 transgenic mice of both sexes | peptid vaccine | synthetic HTLV-1 peptides mixed with the lipohexapeptide N-palmitoyl-S-[2,3-bis(palmitoyloxy)propyl]cysteinyl-seryl-lysyl-lysyl-lysyl-lysine, which is a biocompatible, Th -epitopeindependent adjuvant | 100 µM | NA | intraperitoneally | NA | N-palmitoyl-S-[2,3-bis(palmitoyloxy)propyl]cysteinyl-seryl-lysyl-lysyl-lysyl-lysine | Peptide binding assay, Flow cytometric analysis, Western blot analysis, Cytotoxic T lymphocyte assay | CTL response in response to 11 of 37 tested HLA-B*3501 binding peptides after 3 in vitro stimulations, peptide-specific CTL induction in response to 7 peptides derived from env-gp46 (VPSPSSTPLL, VPSSSSTPL, YPSLALAPH, and YPSLALAPA), pol (QAFPQCTIL), gagp19 (YPGRVNEIL), and tax (GAFLTNVPY) proteins, Bulk CTL generation by four peptides derived from env-gp46 (SPPSTPLLY, VPSPSSTPLLY, and VPSPSSTPLL) and pol (QAFPQCTILQY) killing peptide-pulsed and recombinant vaccinia-infected target cells |
| Fujisawa | 2015 | Japan | in vivo | NA | HTLV-1-infected humanized mouse model (hu-NOG) mouce | tax-peptid vaccine | Mixture of twelve overlapping peptides of 40-42 amino acids long encompassing whole Tax protein | NA | NA | subcutaneously | vaccine was inoculated subcutaneously three times weekly to hu-NOG mouse and then -irradiated HTLV-1 producing Jurkat cells were intraperitoneally injected to infect HTLV-1 | NA | NA | leukemia suppression, retardation of the out growth of human lymphocytes in response to Tax-immunization after HTLV-1 infection, survival of two out of five mice with alimited number of infected T-cells, IL-12 induction and enhanced expression of Tax-specific CD8 T-cell in immunized mice before infection |
| Lucchese | 2021 | Germany | in vitro | NA | NA | mRNA and Peptide-Based Vaccines | Epitope Platform | NA | NA | NA | NA | NA | NA | An epitope platform for HTLV-1 vaccine have been presented to reduce post-vaccination adverse events, cross-reactivity with human antigens |
